# Anaplastic thyroid cancer: Genetic roles, targeted therapy, and immunotherapy

**DOI:** 10.1016/j.gendis.2024.101403

**Published:** 2024-08-30

**Authors:** Zhao Zou, Linhong Zhong

**Affiliations:** a Division of Cardiology, The First Affiliated Hospital of Chongqing Medical University, Chongqing 400010, China; b Chongqing Key Laboratory of Ultrasound Molecular Imaging, Institute of Ultrasound Imaging and Department of Ultrasound, The Second Affiliated Hospital of Chongqing Medical University, Chongqing 400010, China

**Keywords:** Anaplastic thyroid cancer, ATC, Genetic roles, Immunotherapy, Targeted therapy

## Abstract

Anaplastic thyroid cancer (ATC) stands as the most formidable form of thyroid malignancy, presenting a persistent challenge in clinical management. Recent years have witnessed a gradual unveiling of the intricate genetic underpinnings governing ATC through next-generation sequencing. The emergence of this genetic landscape has paved the way for the exploration of targeted therapies and immunotherapies in clinical trials. Despite these strides, the precise mechanisms governing ATC pathogenesis and the identification of efficacious treatments demand further investigation. Our comprehensive review stems from an extensive literature search focusing on the genetic implications, notably the pivotal MAPK and PI3K-AKT-mTOR signaling pathways, along with targeted therapies and immunotherapies in ATC. Moreover, we screen and summarize the advances and challenges in the current diagnostic approaches for ATC, including the invasive tissue sampling represented by fine needle aspiration and core needle biopsy, immunohistochemistry, and ^18^F-fluorodeoxyglucose positron emission tomography/computed tomography. We also investigate enormous studies on the prognosis of ATC and outline independent prognostic factors for future clinical assessment and therapy for ATC. By synthesizing this literature, we aim to encapsulate the evolving landscape of ATC oncology, potentially shedding light on novel pathogenic mechanisms and avenues for therapeutic exploration.

## Introduction

The incidence of thyroid cancer (TC) has experienced a global surge over the past five decades.^1^ Up to 2020, TC is the ninth most prevalent cancer worldwide.^2^ The global age-standardized incidence rate in women (10.1 per 100,000 women) is 3-fold than that in men (3.1 per 100,000 men), while both sexes share a similar global age-standardized mortality rate (0.3 per 100,000 men and 0.5 per 100,000 women, respectively).^2^^,^^3^ Among thyroid malignancies, anaplastic TC (ATC), characterized as the most aggressive form, represents approximately 1.3%–9.8% of cases.^4^ Despite its clinical significance, the underlying mechanisms orchestrating ATC pathogenesis remain enigmatic.

Genetic alterations play a pivotal role in the pathogenesis of ATC. Notably, copy-number aberrations and single-nucleotide variants in ATCs surpass those observed in papillary thyroid cancer (PTC) but are fewer compared with most other adult cancer types.^5^ Patients exhibiting a lower mutation rate (>10 single-nucleotide variants per megabase) demonstrated significantly improved survival (hazard ratio/HR = 0.51; 95% confidence interval/CI: 0.33–0.77; *P* = 0.002).^5^ Common genomic features shared between ATC and differentiated thyroid cancer (DTC) also suggest a common evolutionary origin.^5^ During the anaplastic transformation of DTC, four distinct types of ATC cells emerge, including stress-responsive DTC cells, inflammatory ATC cells, mitotic-defective ATC cells, and mesenchymal ATC cells.^6^ Crucially, two stages are identified in this transformation, the diploid stage, characterized by inflammatory ATC cells exhibiting diploid genomes and inflammatory phenotypes, and the subsequent aneuploid stage, marked by the acquisition of aneuploid genomes and mesenchymal phenotypes by mesenchymal ATC cells.^6^

Recently, advancements in high-throughput sequencing have unveiled significant genetic alterations linked to ATC's development (detailed in Table 1), providing pivotal insights into potential target therapies aligned with these genetic aberrations. These genetic cues have sparked a ray of hope for ATC patients, offering prospects for novel target therapies and immunotherapies.

**Table 1:** Most frequently altered genes detected in cohorts of anaplastic thyroid cancer cases.

Reference	Cases	Gene mutations								
		ALK (%)	BRAF (%)	CKI (%)	EIF1AX (%)	PIK3CA (%)	PTEN (%)	RAS (%)	TERT (%)	TP53 (%)
Glenn et al^274^	7	0	29	NA	0	0	0	14	NA	43
Audrey et al^402^	9	0	67	NA	NA	33	11	0	NA	78
Jeon et al^403^	11	0	91	NA	0	18	9	9	NA	73
Naveen et al^404^	14	0	18	NA	0	18	18	18	36	55
Kunstman et al^103^	22	0	27	9	14	8	0	27	NA	27
Duan et al^102^	25	NA	56	NA	NA	44	NA	28	56	60
Seong-Keun et al^112^	27	0	41	22	33	11	7	44	56	48
Zhang et al^101^	29	NA	24	14	NA	24	NA	14	21	48
Latteyer et al^405^	30	20	7	NA	NA	NA	NA	23	NA	60
Landa et al^20^	33	0	45	0	9	18	15	24	73	73
Khan et al^13^	90	2	34	52	NA	12	13	26	32	66
Vera et al^406^	118	0	11	17	NA	12	0	20	73	55
Xu et al^15^	126	3	45	29	14	18	14	24	75	63
Benjamin et al^105^	144	1	14	3	NA	6	9	43	54	54
Pozdeyev et al^22^	196	1	41	35	NA	14	11	27	65	65
Wang et al^104^	202	NA	42	4	NA	13	8	22	37	59

Note: NA, not accessed; ALK, anaplastic lymphoma kinase; BRAF, v-raf murine sarcoma viral oncogene homolog B1; CKI, cyclin-dependent kinases inhibitor; EIF1AX, eukaryotic translation initiation factor 1A; PIK3CA, phosphatidylinositol-4,5-bisphosphate 3-kinase catalytic subunit alpha; PTEN, phosphatase and tensin homologue; RAS, rat sarcoma oncogene; TERT, telomerase reverse transcriptase; TP53, tumor suppressor gene p53.

In this review, we aim to explore these genetic pointers and their relevance to emerging target therapies and immunotherapies in the context of ATC.

### MAPK signaling pathway

#### Activation and function

Receptor tyrosine kinases (RTKs), integral single-span transmembrane receptors, encompass a diverse spectrum organized into 19 distinct families. Notable among these are epidermal growth factor receptor (EGFR), platelet-derived growth factor receptor (PDGFR), vascular endothelial growth factor receptor (VEGFR), and fibroblast growth factor receptor (FGFR).^7^ Each RTK family binds to specific extracellular ligands, initiating intracellular signaling cascades upon binding.^7^

Upon activation, RTKs operate upstream of rat sarcoma (RAS), a small GTPase comprising three gene isoforms: HRAS, NRAS, and KRAS.^8^ RAS proteins directly engage phosphatidylinositol 3-kinase (PI3K), catalyzing the conversion of phosphatidylinositol 4,5-biphosphate (PIP_2_) into phosphatidylinositol 3,4,5-trisphosphate (PIP_3_).^9^ Consequently, RAS exerts its influence upstream in both the MAPK and PI3K-AKT signaling pathways.

Activated RAS interfaces with rapidly accelerated fibrosarcoma (RAF), encompassing three isoforms: raf-1 proto-oncogene, serine/threonine kinase (CRAF), v-raf murine sarcoma viral oncogene homolog B1 (BRAF), and a-raf proto-oncogene, serine/threonine kinase (ARAF).^10^ RAF heterodimerizes with MAPK kinase (MEK), and active MEK phosphorylates extracellular signal-regulated kinase (ERK).^10^ Subsequently, ERK travels to the nucleus, modulating various transcription factors through phosphorylation.^11^ This MAPK signaling pathway is typically associated with cellular proliferation and survival mechanisms.^12^

### Role of MAPK signaling pathway in ATC

#### RAS

RAS mutations manifest as a prevalent occurrence in ATC. These mutations span across all RAS isoforms,^8^ with NRAS mutations exhibiting a higher incidence among patients younger than 50 years.^13^ Notably, RAS mutations are less frequent in secondary ATC compared with primary cases and are frequently linked to unfavorable prognoses.^14^ Cases displaying BRAF or RAS mutations demonstrate similar frequencies in nodal and distant metastases.^15^^,^^16^ Of significance, KRAS^G12D^, in conjunction with thyroid hormone receptor beta (THRβ), orchestrates myc up-regulation, hastening ATC progression.^17^ Patients harboring wild-type KRAS codon 12/13 exhibited a median overall survival (mOS) of 19 weeks.^18^

Distinct associations surface between RAS mutations and other gene alterations. For instance, a mutual exclusivity is observed between BRAF^V600E^ and RAS alterations.^13^ Moreover, RAS mutations and tumor suppressor gene p53 (TP53) mutations dominate and exhibit mutual exclusivity in ATC and poorly differentiated thyroid cancer (PDTC).^19^ Additionally, a gradually emerging correlation between eukaryotic initiation factor IAX (EIFIAX) mutations and RAS in ATC is evident. EIF1AX mutations frequently co-occur with RAS mutations in 117 PDTC and ATC cases.^20^ Notably, within a cohort of 31 patients, all three cases exhibiting combinations of several genetic mutations (EIF1AX, RAS, TERT, and TP53) were diagnosed as ATC.^21^

Proposals have emerged for a standardized classification of ATC based on RAS and other molecular biomarkers. This includes delineating type 1 ATC (BRAF-positive), likely originating from PTC; type 2 ATC (NRAS-positive), potentially originating from follicular thyroid cancer (FTC); type 3 ATC (mutated RAS-positive), potentially originating from FTC or Hürthle cell carcinoma; and a mixed ATC subtype characterized by inactive mutations in cell-cycle regulation genes (*e.g.*, CDKN2A and CDKN2B).^22^

#### RAF (BRAF, BRAF^V600E^)

The role of BRAF mutation, particularly BRAF^V600E^, emerges as pivotal in ATC pathogenesis. Encoded by the BRAF^T1799A^ mutation,^23^ BRAF^V600E^ drives heightened extracellular signal-regulated kinase phosphorylation, fostering aberrant cell proliferation and stifling the essential genes crucial for radioiodine responsiveness in TC.^24^ B-cell lymphoma-2-associated athanogene 3 (BAG3) interaction with BRAF prevents proteasome-mediated degradation, sustaining ATC cell growth.^25^ Notably, *in vivo* experiments demonstrated that silencing BRAF inhibited tumor growth.^26^ Outcomes were notably worse in cases displaying concomitant BRAF/RAS and TERT mutations compared with singular mutations,^15^ with BRAF mutations exhibiting greater prevalence in secondary ATC.^14^ Consequently, BRAF status assessment became a staple in ATC evaluations, with immunohistochemical detection showcasing 100% sensitivity and 95.7% specificity for BRAF^V600E^ status in ATC.^27^ Additionally, reports indicate the utility of droplet digital PCR, based on fine needle aspiration (FNA), for rapid BRAF^V600E^ detection in unresectable ATC.^28^

BRAF^V600E^ significantly accelerates ATC progression, orchestrating cellular lactylation to promote proliferation.^29^ It collaborates with PIK3CA^H1074R^ (Phosphatidylinositol-4,5-bisphosphate 3-kinase catalytic subunit alpha (PIK3CA) is one of PI3K catalytic subunits) or silences PTEN to advance ATC pathogenesis.^30^ Moreover, it impedes mitochondrial permeability transition through the pERK-pGSK-CypD pathway, thwarting ATC cell death.^31^ Activation of the JAK/STAT pathway in BRAF^V600E^ ATC cells contributes to resistance against BRAF inhibitors.^32^ Knockdown of S100A4, overexpressed in ATC, led to reduced BRAF^V600E^ expression, curbing proliferation and metastasis.^33^ Interestingly, BRAF^V600E^ correlates with cell-free DNA markers ALU83 and ALU244, associated with increased methylation, albeit detected less frequently in ATC than in PTC,^34^ linking BRAF^V600E^ to oncogenic hypermethylation.^35^

Additionally, BRAF-mutated ATC demonstrates a robust association with PTC. Evidence suggests both BRAF-positive PDTC and ATC harbor regions of preexisting papillary carcinoma, affirming BRAF mutations in well-differentiated and dedifferentiated components.^36^ The presence of BRAF mutation, including BRAF^V600E^, is observed in ATC coexisting with PTC.^37^, ^38^, ^39^, ^40^ Notably, cases of BRAF mutation were identified in ATCs derived from BRAF-mutant PTCs,^41^ underscoring the engagement of BRAF mutation, particularly BRAF^V600E^, in the tumorigenesis of both ATC and PTC.

### PI3K-AKT-mTOR signaling pathway

#### Activation and function

The intertwined signaling pathways of PI3K-AKT and the mechanistic target of rapamycin (mTOR) stand as pivotal regulators orchestrating cell growth and survival within a unified signal axis.^42^ PI3K catalyzes the conversion from PIP_2_ to PIP_3_, where PIP_3_ recruits protein kinase B (AKT) to the cellular membrane.^43^ AKT, in turn, governs cell survival and proliferation while exerting a positive regulatory effect on mTOR.^43^ mTOR elevation contributes to increased levels of tumorigenesis-associated proteins like hypoxia-inducible factor^44^ and cyclin D1.^45^ Active AKT demonstrates heightened nuclear distribution and expression levels in both ATC and PTC. AKT deficiency correlates with diminished cellular proliferation and invasive potential, underscoring its role in disease progression.^46^ Notably, PTEN, a pivotal downstream effector of the PI3K-AKT-mTOR pathway, functions as a protein and lipid phosphatase. Its role involves the dephosphorylation of PIP_3_ into PIP_2_,^47^ thereby inhibiting the PI3K-AKT-mTOR cascade. By modulating PIP_3_ levels, PTEN intricately regulates cell survival, proliferation, and migration.^48^ Moreover, the PI3K-AKT pathway potentially operates downstream of the centrosomal protein of 55 kDa (CEP55), an independent prognostic indicator in ATC.^49^ This suggests a regulatory relationship between CEP55 and the PI3K-AKT pathway, further emphasizing the intricate interplay of molecular mechanisms influencing ATC progression and prognosis.

### Role of PI3K-AKT-mTOR signaling pathway in ATC

#### Prevalence of mutations in ATC

Mutations within the PI3K-AKT-mTOR signaling pathway emerge as frequently observed in ATC. Notably, mutations in PIK3CA, AKT, and PTEN are more prevalent in ATC compared with FTC.^50^ In a subset of PIK3CA-mutant ATC cases, activation of AKT was evident in 9 out of 16 instances.^51^ Within a cohort of 50 ATC cases, rates of PIK3CA copy gain, PIK3CA mutations, and PTEN mutations stood at 42%, 12%, and 16%, respectively.^52^ The concurrent presence of BRAF and PIK3CA mutations in ATC was reported at a rate of 10.3%,^53^ both identified as adverse prognostic factors for ATC patient survival.^54^ Notably, patients exhibiting a PIK3CA mutation detected in circulating cell-free DNA showcased poorer overall survival (OS).^55^ Recent findings revealed a novel mTOR point mutation (A1256G, exon 9) identified in the C643 ATC cell line.^56^ Moreover, a comprehensive evaluation across 14 ATC cases unveiled a complete loss of PTEN mRNA expression in 4 instances, correlating significantly with the anaplastic subtype.^57^ Transcriptional silencing of PTEN emerges as a noteworthy association within the context of ATC pathology, emphasizing its potential role in disease progression and subtype delineation.

#### Development of ATC

The migration and invasion of ATC cells exhibit strong correlations with the status of the PI3K-AKT-mTOR pathway. O-GlcNAcylation significantly augments ATC cell invasion, partly attributed to PI3K-AKT signaling.^58^ MicroRNA-125b exerts inhibitory effects on tumor migration and invasion by targeting phosphoinositide 3-kinase catalytic subunit delta (PIK3CD), an alternate PI3K catalytic subunit.^59^ Within the intricate network, the PI3K-AKT pathway interconnects with various axes influencing ATC aggressiveness. For instance, the HOXD9-MicroRNA-451a-PSMB8 axis modulates apoptosis, promotes epithelial–mesenchymal transition, and exacerbates metastasis within ATC, all orchestrated via the PI3K-AKT signaling cascade.^60^ Vascular cell adhesion molecule-1 (VCAM-1) contributes to migration and invasion through the PI3K-AKT-mTOR pathway *in vitro*, with both VCAM-1 and the pathway showing activation in BRAF-inhibition treatment resistance.^61^ Moreover, SrY-related HMG box-2 (SOX2) intensifies ATC aggressiveness via PI3K-AKT-mediated fibronectin 1 (FN1) up-regulation.^62^ The insulin-like growth factor (IGF) produced by M2-like tumor-associated macrophages (TAMs) augments ATC stemness and invasion by activating the IR-A/IGF1R-mediated PI3K-AKT-mTOR pathway.^63^ Intriguingly, grb2-associated binder 1 (GAB1) up-regulation stimulates AKT activation, cellular migration, and invasion through AKT-MDR1,^64^ while GANT61 suppresses invasion and epithelial–mesenchymal transition by targeting AKT-mTOR or JAK-STAT3 pathways in ATC.^65^ A-kinase interacting protein 1 (AKIP1) knockdown inhibits PI3K-AKT and β-catenin pathways, mitigating cell invasion and reinstating sensitivity to doxorubicin (DOX).^66^ These multifaceted interactions underscore the intricate role of the PI3K-AKT-mTOR pathway in dictating ATC aggressiveness and therapeutic responses.

#### Resistance to agents

The PI3K-AKT-mTOR pathway significantly contributes to chemotherapy resistance in ATC. Strategies targeting this pathway have shown promise in overcoming resistance mechanisms. Lexatumumab, acting as a TNF-related apoptosis-inducing ligand receptor 2 (TRAIL-R2) agonist antibody, effectively circumvented resistance to apoptosis by inhibiting BRAF^V600E^, PI3K, and MAPK.^67^ In BRAF^V600E^-mutant ATC cells, c-Met-mediated reactivation of the PI3K-AKT and MAPK pathways substantiates resistance to vemurafenib, an effect mitigated through the dual inhibition of BRAF and c-Met.^68^ Similarly, concurrent inhibition of Src Family Kinases (Src) and MAPK circumvented resistance to dasatinib, a dual Src/Bcr-Abl inhibitor, by impeding PI3K-AKT pathways *in vitro*.^69^ Furthermore, the combination of PI3K-mTOR inhibition potentiated the reversal of resistance to palbociclib, a CDK4/6 inhibitor, in ATC.^70^ These approaches illustrate the potential of targeting the PI3K-AKT-mTOR pathway to overcome chemotherapy resistance in ATC, offering new avenues for therapeutic intervention.

### Interaction with other proteins

#### Forkhead-box (FOX) family proteins

The involvement of forkhead-box (FOX) family proteins in the PI3K-AKT-mTOR pathway of ATC underscores their significance in disease progression. Forkhead box protein A1 (FOXA1) exhibits heightened expression levels in ATC, and its suppression results in G_1_ growth arrest and reduced cell proliferation.^71^ Conversely, forkhead box protein M1 (FOXM1) displays substantial up-regulation in ATCs compared with normal thyroid tissue and other TC types. Elevated FOXM1 levels correlate with TP53 loss-of-function and hyperactivation of the PI3K-AKT-FOXO3a pathway.^72^ Inhibiting FOXM1 proves effective in reducing tumor burden and curbing metastasis in ATC.^72^ Furthermore, FoxO3a, a pivotal regulator in tumor growth, undergoes AKT-mediated phosphorylation, leading to its exclusion from the nucleus. In its non-phosphorylated state at S473, FoxO3a remains within the nucleus, promoting ATC proliferation by transcriptionally up-regulating cyclin A1.^73^ Forkhead box K2 (FOXK2) orchestrates the transcriptional activation of vascular endothelial growth factor A (VEGFA), which, upon binding to VEGFR1, triggers ERK, PI3K-AKT, and P38-MAPK signaling pathways, fostering angiogenesis.^74^ This angiogenic process contributes to resistance against apatinib, a VEGFR2 inhibitor, in ATC.^74^ The intricate involvement of FOX family proteins in ATC underscores their multifaceted roles in disease progression and therapeutic resistance.

#### Sodium-iodide symporter

Sodium iodide symporter (NIS), a pivotal plasma membrane glycoprotein, serves as the conduit for iodide transportation into the thyroid.^75^ In radioactive iodine (^131^I) therapy for TC, NIS facilitates the uptake of radioactive iodine into TC cells, effectively impeding tumor growth.^76^

Experimental work has recently gradually removed the veil over the relationship between NIS and the PI3K-AKT-mTOR axis. *In vitro* studies demonstrated that dual inhibition of MEK or BRAF^V600E^ and PI3K resulted in an up-regulation of NIS expression.^77^ Similarly, *in vivo*, MEK inhibition exhibited an up-regulatory effect on sodium-iodine symporter expression.^77^ The intricate regulation of NIS involves the PI3K-AKT-mTOR signaling pathway, as evidenced by its modulation by CTOM-DHP, leading to endogenous NIS up-regulation concomitant with the inhibition of PI3K-AKT and MAPK signaling pathways in 8505C ATC cell line.^78^ Noteworthy associations emerged between the expression levels of NIS and PTEN and the grade of TC differentiation.^79^ Moreover, in resveratrol-suppressed ATC cells, significant features including PTEN up-regulation and concurrent nuclear translocation of NIS and PTEN were observed.^79^ This intricate interplay highlights the multifaceted regulatory network governing NIS expression and its correlations with the PI3K-AKT-mTOR signaling pathway.

Noteworthily, targeting NIS stands as a challenging target in managing ATC. Radio-active iodine therapy showed its paralysis on the ATC due to the cellular resistance to radio-iodine originating from the NIS absence or down-regulation.^80^ There are three innovative approaches for improving the radiotoxicity of ATC, namely nanoparticles, agents, and viruses. i) Nanoparticles. Several novel nanoparticles have been developed to ameliorate ATC's radiotoxicity, like human serum albumin (HSA)-MnO_2_, mesoporous silica nanoparticles, and tyrosine–hyaluronic acid–polyethyleneimine.^80^ The lipid-peptide-mRNA nanoparticles experimentally smoothed the radio-iodine therapy for ATC significantly.^81^ Combination therapy of ^131^I and other agents (cerebroid polydopamine and indocyanine green) improved the therapeutic effect on ATC.^80^ Radio-sensitization of Prima-1, a TP53 mutant restoring agent, enhances the therapeutic impact of ^131^I-labelled nanoparticles by reactivating mutant TP53.^82^ ii) Agents. Tunicamycin enhanced the ATC redifferentiation and radio-active iodine uptake by rescuing the NIS expression,^83^ and bortezomib facilitated the iodide accumulation and showed a therapeutic effect on ATC.^84^ Autophagy-activating digitalis-like compounds increased the expression of thyroglobulin and ^131^I uptake by restoring NIS significantly,^85^ and targeting estrogen-related receptor γ (ERRγ) by its inverse agonists brought the improvement of NIS function via MAPK signaling pathway in ATC cells.^86^, ^87^, ^88^ Tissue factor (TF) presence was notably high in the THJ-16T ATC cell line, and the combination of two TF-specific agents (^64^Cu-NOTA-ALT-836 and ^131^I-ALT-836) demonstrated efficacy in managing ATC *in vivo*.^89^ Furthermore, ^131^I-labeled caerin 1.1 peptide showcased inhibition of ATC tumor growth and migration.^90^ iii) Virus. One vaccinia virus, GLV-1h153, is an oncolytic agent against ATC by promoting radio-iodine uptake.^91^ Measles virus-mediated NIS expression has shown its therapeutic effect on ^131^I-resistant ATC,^92^ and adenoviral transfer of NIS exhibited its best performance in increasing NIS expression and radio-iodine administration at post-transfer day 2.^93^

#### Thyroid hormone receptor β

The association between thyroid hormone receptor β (THRβ) and the PI3K-AKT pathway in ATC has gradually come under scrutiny. Studies indicate that elevated expression of THRβ1 influences differentiation phenotypes and fosters cell proliferation in the ARO ATC cell line.^94^ Conversely, THRβ curtails ATC aggressiveness, promoting apoptosis by inhibiting the PI3K-AKT pathway.^95^ This inhibition is rooted in the down-regulation of receptor tyrosine kinase (RTK) and the concurrent up-regulation of phosphoinositide and AKT phosphatase *in vitro*.^96^ Experiments showcasing selective activation of THRβ have demonstrated tumor-suppressive effects in female mice, underscoring its potential therapeutic significance.^97^ Additionally, THRβ exhibits a restraining effect on the activity of ATC cancer stem cells.^98^^,^^99^ Despite these insights, further investigations are warranted to delineate the precise role of THRβ in the pathology of ATC. Continued research holds promise for a deeper understanding of THRβ′s implications in ATC and its potential as a therapeutic target.

### Other frequent gene aberrations

#### Anaplastic lymphoma kinase

Anaplastic lymphoma kinase (ALK), a receptor tyrosine kinase (RTK), typically governs cell proliferation and survival during nervous system development. Positioned on chromosome 2's short arm (2p23), ALK frequently undergoes chromosomal recombination with other genes (X) to form X-ALK fusion oncoproteins, known as ALK rearrangements.^100^ These fusion oncoproteins activate ALK, thereby contributing to the pathogenesis of ATC.

Despite the exceedingly low prevalence of ALK rearrangements in ATC, their involvement in tumorigenesis remains evident.^101^, ^102^, ^103^, ^104^, ^105^, ^106^ A comprehensive whole-transcriptome analysis highlighted STRN-ALK fusion as the most frequent in TC. Its prevalence was notably higher in PDTC and ATC compared with other well-differentiated forms.^107^ The expression of STRN-ALK, coupled with concurrent TP53 loss, instigates thyroid carcinogenesis, leading to multi-step dedifferentiation progressing from PTC to PDTC and ATC *in vivo*.^108^

Intriguingly, two novel point mutations, C3592T and G3602A, were identified in exon 23 of the ALK gene in ATC.^109^ Both mutations heightened tyrosine kinase activities and facilitated cell invasion.^109^ Despite these findings, the precise role of ALK rearrangements in ATC remains enigmatic, necessitating further research for a comprehensive understanding of their impact on the disease.

#### Cyclin-dependent kinase

Cyclin-dependent kinases (CDKs), a family of serine/threonine kinases, orchestrate distinct phases of the cell cycle in collaboration with cyclins and cyclin-dependent kinase inhibitors (CKIs).^110^ The regulatory action of CKIs, comprising the CDK-interacting protein/kinase inhibitory protein (CIP/KIP) family and the inhibitor of kinase (INK) family, modulates CDK activity. CIP/KIP members such as p21^cip1/waf1^ (CDKN1A, or p21), p27^kip1^ (CDKN1B, or p27), and p57^kip2^ (CDKN1) impede CDK function by disrupting CDK-cyclin interactions, while the INK family encompassing p15^INK4b^ (CDKN2B, or p15), p16^INK4a^ (CDKN2A, or p16), p18^INK4c^ (CDKN2C, or p18), and p19^INK4d^ (CDKN2D) primarily binds to CDKs.^111^

Maintaining the interplay among CDKs, cyclins, and CKIs is crucial for normal cell cycle progression. Mutations affecting CDKs and their partners, particularly CKIs, have been implicated in the initiation and progression of ATC. Copy number losses and mutations in CDKN2A and CDKN2B have shown associations with ATC.^22^

ATC exhibited higher mutation rates in TP53 and CDKN2A compared with advanced DTC, with CDKN2A loss significantly correlating with poorer disease-specific survival in ATC or advanced DTC cases.^112^ In ATC cases, CDKN2A mutations, either from copy number loss or truncating mutations, were detected in 5 out of 8 cases, often concurrent with CDKN2B loss, resulting in diminished mRNA expression of both genes.^113^

Two key CKIs, CDKN1A and CDKN1B, have shown a profound association with restricting ATC cell proliferation. CDKN1A augmented apoptosis when combined with manumycin and paclitaxel (PTX) *in vitro*.^114^ Intriguingly, both CDKN1A and CDKN1B were up-regulated in cell cycle arrest induced by diverse agents like bone morphogenetic protein (BMP-7),^115^ butyrate,^116^ simvastatin,^117^ and insulin-like growth factor binding protein 7 (IGFBP7).^118^ However, further exploration is warranted to elucidate the precise roles of CDKN1A and CDKN1B in constraining ATC cell proliferation.

#### Telomerase reverse transcriptase

Telomeres, situated at chromosomal ends, undergo gradual shortening during DNA replication, a process significantly contributing to cellular senescence. To counteract this shortening, telomerase, composed of telomerase RNA (the lengthening template) and telomerase reverse transcriptase (TERT, the catalytic subunit), elongates excessively shortened telomeres, enabling DNA replication and averting cellular senescence.^119^ Activation of TERT due to mutations immortalizes TC cells, and its underlying mechanisms have been progressively elucidated.

TERT mutations are notably prevalent in ATC, ranging from 21% to 75%.^13^^,^^15^^,^^101^^,^^102^^,^^104^ In a cohort of 106 American and Chinese ATC samples, the frequency of the TERT 1,295,228 C > T (TERT^C228T^) mutation was 34.9% (37 samples), showing an association with older patient age (*P* = 0.02).^120^ Notably, TERT promoter mutations, especially C228T, tended to co-occur with BRAF^V600E^ mutation.^121^

These TERT mutations, particularly in the promoter region, are correlated with poorer prognoses. Within the previously mentioned cohort, a robust association was observed between TERT^C228T^ and distant metastasis in the American subset.^120^ Long-term survivors of ATC (alive for 2 years or longer) exhibit lower rates of concurrent RAS/BRAF and TERT promoter mutations compared with ATC control cases.^122^ Furthermore, TERT promoter mutations strongly correlate with increased clinical burden and an unfavorable prognosis.^121^ Independently, TERT promoter mutation is linked with the anaplastic transformation of papillary carcinoma.^123^ Recurrent papillary carcinomas with anaplastic transformation showcase a higher prevalence of BRAF^V600E^ mutation and TERT promoter mutation compared with those without anaplastic transformation.^124^ These findings highlight the intricate associations between TERT mutations and the clinical outcomes in ATC.

#### Tumor suppressor gene P53

Wild-type TP53 serves critical roles in arresting the cell cycle, aiding in DNA repair, and triggering apoptosis when confronted with DNA damage.^125^ However, in various human cancers, TP53 mutations strip it of these essential functions.^126^

In the context of TC, TP53 mutations exhibit a significant correlation with ATC compared with other types. ATC showcases substantial TP53 up-regulation in contrast to PTC.^127^ TP53 overexpression is prevalent in anaplastic carcinoma but not in insular carcinoma, suggesting its involvement in dedifferentiating from insular thyroid carcinoma to ATC.^128^ Additionally, distinct TP53 mutation patterns between FTC and ATC lesions further indicate the specificity of TP53 mutations in ATC progression.^129^ Notably, TP53-mutated adenomas may represent precursors for ATC, albeit in a limited proportion.^130^ Homozygous phenotypes at codon 72 of TP53 have been identified as potential risk factors for developing ATC.^131^

The co-occurrence of TP53 mutations with other genetic alterations is common in ATC. Such combinations, like frameshift insertions in PTEN and TP53, have been associated with brain metastasis.^132^ TP53 and TERT mutations are more frequent in ATC compared with angiosarcoma and PDTC,^133^^,^^134^ and TP53 mutation was associated with a shorter survival time.^133^

The precise role of TP53 mutations in ATC remains elusive. Presently, two primary aspects shed light on this role: i) the functional status of TP53 mutations appears to influence the response of ATC cells to evodiamine-induced apoptosis and G_2_/M arrest.^135^ Gain-of-function TP53 mutations have been linked to increased galectin-3 expression, fostering chemoresistance in ATC.^136^ Conversely, loss of TP53 function seems to facilitate the transition from BRAF^V600E^-harboring PTC to ATC *in vivo*.^137^ In comparison to PTC, ATC exhibits higher levels of α-l-fucosidase-1 (FUCA1), regulated in part by TP53 status, and lower levels of fucosyltransferase-8, resulting in elevated fucose levels on cell surface glycans, contributing to ATC aggressiveness.^138^ ii) TP53 displays intricate interactions with various proteins in ATC. It plays a role as a functional interactor of SOX2, influencing ATC stemness regulation.^139^ Mutant TP53 (G199V) was observed to enhance resistance to apoptosis by suppressing STAT3 in the KAT-18 ATC cell line.^140^ Notably, TP53 overexpression counteracts the heightened expression of mini-chromosome maintenance protein 7 (MCM7), which is closely associated with tumor malignancy in ATC.^141^ Furthermore, *in vitro* studies indicate that decreased junctional adhesion molecule A (JAM-A) levels in ATC alleviate aggressiveness through the phosphorylation of TP53 and GSK3α/β pathways.^142^

#### Wnt

The Wnt signaling pathway orchestrates cellular responses via extracellular Wnt signals binding to a co-receptor on the cell membrane, consisting of a frizzled family member and a low-density lipoprotein receptor-related protein (LRP) family member, and intracellular components (destruction complex), including glycogen synthase kinase 3 beta (GSK3β), casein kinase 1 alpha (CK1α), axis inhibition protein (AXIN), and adenomatous polyposis coli (APC).^143^ In the absence of Wnt signals, the destruction complex phosphorylates β-catenin, marking it for ubiquitylation and subsequent proteasomal degradation, thereby deactivating Wnt target genes.^144^ However, upon Wnt signal binding to the co-receptor, inhibition of the destruction complex ensues, elevating β-catenin levels and activating Wnt target genes.^144^

The Wnt signaling pathway is pivotal in regulating adult stem cell homeostasis and tissue regeneration, and has implications in the development of ATC.^145^ Analysis of a Japanese ATC cohort identified mutation frequencies of 4.5% for β-catenin, 9.0% for APC, and 81.8% for AXIN1, with observed overexpression of Wnt target genes, cyclin D1 (27.3%) and c-myc (59.1%).^146^ The abnormal spindle-like microcephaly-associated protein (ASPM) was found to expedite ATC progression by modulating the Wnt/β-catenin signaling pathway.^147^ Additionally, hyper-activation of the Wnt signaling pathway was associated with resistance to artemisinin, which was overcome by the Wnt signaling inhibitor, pyrvinium pamoate.^148^ However, a comprehensive understanding of Wnt signaling's role in ATC remains limited.

Studies involving therapeutic interventions targeting the Wnt/β-catenin pathway in ATC have shown promise. A conditionally replicative adenovirus (named “HILMI”) targeting this pathway demonstrated therapeutic efficacy.^149^ Furthermore, ellagic acid was found to inhibit ATC *in vitro* by impeding the Wnt/β-catenin and PI3K-AKT pathways.^150^ More comprehensive research focusing on targeting the Wnt signaling pathway in ATC treatment is warranted.

#### Mitochondrial metabolism

Abnormalities in mitochondrial metabolism play a pivotal role in the pathogenesis and progression of TC, rendering mitochondrial metabolism an enticing therapeutic target for combatting ATC.^151^ ATC presents distinctive features in mitochondrial metabolism, with two key markers, monocarboxylate transporter 1 (MCT1) and outer mitochondrial membrane member 20 (TOMM20), being significantly up-regulated in ATC compared with non-cancerous thyroid tissue.^152^ Expression of two mitochondrial enzymes, serine hydroxymethyltransferase-2 (SHMT2) and methylenetetrahydrofolate dehydrogenase 2 (MTHFD2), correlates with lower thyroid differentiation scores and adverse clinical outcomes in ATC patients.^153^ Notably, inhibition of SHMT2 disrupts mitochondrial respiration, exhibiting therapeutic potential in ATC treatment.^153^ The hyper-activation of mitochondrial one-carbon metabolism in ATC underscores its significance in nucleotide and glycine synthesis.^154^ Furthermore, compared with PTC or normal thyroid tissue, ATC cells induce the release of neutrophil extracellular DNA traps associated with mitochondrial reactive oxygen species production.^155^ Although the precise role of mitochondrial metabolism in ATC tumorigenesis awaits full elucidation, targeting mitochondrial metabolism offers a novel therapeutic avenue for ATC management.

Two facets of mitochondrial metabolism hold promise in this regard. One is mitochondrial-mediated apoptosis, a focus of intense research as a promising strategy for targeting mitochondrial metabolism. Various agents have demonstrated efficacy in blocking ATC cell proliferation by promoting mitochondrial-mediated apoptosis, including 5F,^156^ diallyl sulphide (DAS),^157^ and niclosamide.^158^ Berberine blocked ATC cell proliferation by inducing mitochondrial-mediated apoptosis and inhibited migration via MAPK and PI3K-AKT signaling pathways,^159^ while diallyl trisulfide (DATS) was found to induce mitochondrial-mediated apoptosis by triggering DNA damage in 8505C ATC cell line.^160^ Capsaicin induces mitochondrial calcium overload and subsequent mitochondrial-mediated apoptosis in ATC cells by targeting transient receptor potential vanilloid type 1 (TRPV1).^161^ Bortezomib (proteasome inhibitor) and TRAIL synergistically inhibited ATC cells by mitochondrial-mediated apoptosis.^162^ The other facet is the mitochondrial membrane potential, emerging as another potential target within mitochondrial metabolism. Two agents, mitotane^163^ and sodium orthovanadate,^164^ impeded ATC cell proliferation by disrupting mitochondrial membrane potential and inducing apoptosis. Silencing of MAPK-associated death domain-containing protein (MADD) correlates with reduced mitochondrial membrane potential,^165^ while a combination of MADD-siRNA and TRAIL exhibits therapeutic efficacy in TRAIL-resistant ATC models.^166^

Additionally, three mitochondrion-target agents aimed at other aspects of mitochondrial metabolism, artemisinin, artesunate, and ruxolitinib, are hopeful. Artemisinin inhibits the mitochondrial respiratory chain proteins in CAL-62 and BHT-101 ATC cell lines, and CAL-62 cells show drug resistance to artemisinin by blocking pyruvate dehydrogenase.^167^ Artesunate, the derivative of artemisinin,^168^ blocks growth and promotes apoptosis in chemo-resistant KAT-4 ATC cells by impeding mitochondrial functions without affecting glycolysis and acts synergistically with DOX.^169^ Ruxolitinib promoted apoptosis and pyroptosis in ATC by blocking dynamin-related Protein 1 (DRP1)-mediated mitochondrial fission.^170^ More comprehensive research focusing on targeting mitochondrial metabolism in ATC treatment is warranted.

Targeting mitochondrial metabolism also amplifies the efficacy of chemotherapy for ATC. Atovaquone significantly augments the anti-proliferative effect of DOX *in vitro* by blocking mitochondrial respiration and STAT3,^171^ and combined treatment with octreotide and cisplatin blocked proliferation and induced mitochondrial-mediated apoptosis in the side population cells of ATC.^172^ Besides, the combination of photodynamic therapy and carboplatin shows a therapeutic effect on ATC by breaking mitochondrial metabolism. The combination of photodynamic therapy and carboplatin synergistically enhanced mitochondrial membrane depolarization and induced mitochondrial-mediated apoptosis in the FRO ATC cell line.^173^ *In vivo* studies validated the synergistic effect of carboplatin and photodynamic therapy on mitochondrial metabolism and showed that this combination blocked the expression of EGFR and PI3K while activating PTEN.^174^ Further investigations are warranted to fully elucidate the value of targeting mitochondrial metabolism in enhancing the efficacy and safety of chemotherapy for ATC.

### Targeted therapy for ATC

#### Effect and limit of trimodal therapy

All ATC cases are categorized as TNM stage IV (IVA-IVC).^175^ The standard treatment, known as trimodal therapy, encompasses surgery, chemotherapy, and external beam radiotherapy.^175^

Trimodal therapy stands as a cornerstone in combatting ATC. Patients undergoing multimodal therapy experienced a prolonged mOS of 21 months (range, 5.8–44), distinctly surpassing those on palliative therapy (mOS, 3.9 months; range, 2.7–5.3) (HR = 0.32; *P* = 0.0006).^176^ Aggressive multimodal therapy led to a remarkable 60-month mOS for patients with locoregionally confined ATC, with 50% of cases alive and free from cancer (follow-up duration >32 months).^177^ When compared, patients subjected to trimodal treatment demonstrated an extended mOS of 22.1 months, surpassing those with dual therapy involving external beam radiotherapy and chemotherapy (mOS = 6.5 months; *P* = 0.0008).^178^ Radiotherapy doses ≥60 Gy correlated with improved locoregional progression-free survival (HR = 0.135; *P* = 0.001) and OS (HR = 0.487; *P* = 0.004), while trimodal therapy itself was linked to enhanced locoregional progression-free survival (HR = 0.060; *P* = 0.017).^179^

However, trimodal therapy reveals limitations across different stages of ATC. Conventional chemotherapy and radiation therapy yield no added benefit for most stage IVA patients but do extend survival for IVB patients.^180^ The difference in mOS between the multimodal and palliative therapy groups was notable in stage IVB patients (22.4 *vs*. 4 months; HR = 0.12; 95%CI: 0.03–0.44; *P* = 0.0001), but not in stage IVC (HR = 1.15; 95%CI: 0.4–3.2; *P* = 0.78).^176^ Trimodal therapy prolonged the mOS of stage IVA/B patients compared with surgery alone (25 *vs*. 3 months; *P* = 0.04), yet it exhibited no significant effect on the mOS of stage IVC patients compared with debulking procedures (6 *vs*. 7 months; *P* = 0.25).^181^ While trimodal therapy improves the survival of regionally confined ATC patients, it cannot effectively control advanced metastatic ATC.^182^ Challenges in trimodal therapy will be discussed later.

Due to the constraints of trimodal therapy, targeted therapy has progressively emerged as a crucial aspect of ATC treatment. The activation of either RAS-MAPK-ERK or PI3K-AKT-mTOR pathway is mutually exclusive in ATC, and inhibiting either pathway enhances sensitivity to chemotherapy.^183^ Dual inhibition of BRAF^V600E^ and MEK significantly reduces tumor size, extent of surgery, and surgical morbidity score.^184^ Targeted therapy is associated with a favorable OS, and the combination of surgery, radiotherapy, and targeted therapy (mOS = 34.3 months; 6-month survival rates = 77.8%) proved most effective.^185^ Factors linked to improved OS include targeted therapy (HR = 0.49; 95%CI: 0.39–0.63; *P* < 0.001), immunotherapy combined with targeted therapy (HR = 0.58; 95%CI: 0.36–0.94; *P* = 0.03), and surgery accompanied by BRAF-targeted therapy (HR = 0.29; 95%CI: 0.10–0.78; *P* = 0.02).^186^ The discussion on advances in targeted therapy for ATC will follow.

#### Overview of current ATC targeting therapy and immunotherapy

In the current treatment guideline for ATC, targeted therapy was recommended for stage IVB-IVC patients.^175^ For stage IVB cases, dabrafenib and trametinib were recommended as the BRAF^V600E^ mutation was detected. This combination granted patients for further trimodal therapy. The therapeutic flow of stage IVC cases shared this solution with stage IVB cases when carrying BRAF^V600E^ mutation, and immunotherapy targeting PD-1/PD-L1 was recommended under the high PD-L1 expression or tumor mutational burden higher than ten mutations. However, except dabrafenib–trametinib combination, the recommended clinical application of agents targeting MAPK and PI3K-AKT-mTOR signaling pathway was still limited, and immunotherapy was ranked in the conditional strength of recommendation with the low quality of evidence.

The following sections will outline the advances in ATC targeted therapy and immunotherapy from dabrafenib to other candidate agents in detail. We found that clinical studies and case reports were mainly concerned with the results of targeting MAPK signaling pathway and RAF (especially BRAF^V600E^), like lenvatinib, dabrafenib, and dabrafenib-trametinib, and immunotherapy targeting PD-1/PD-L1. Reports about drugs aimed at the PI3K-AKT-mTOR signaling pathway and other targets mainly showed their therapeutic potential experimentally, and more clinical steps were warranted for their efficacy and safety. Additionally, the global ongoing clinical trials for ATC treatment were also collected and listed in Table 7. These clinical trials can provide more candidate agents for improving the future prognosis and life quality of ATC patients.

**Table 3:** Efficacy and safety results from clinical studies of other target therapies for anaplastic thyroid cancer.

Agents	Design	Cases	PR (%)	CR (%)	SD (%)	PD (%)	mPFS	mOS	Adverse events	Reference
Dabrafenib + trametinib	Retrospective study	17	70.6	11.8	0	17.6	4.7 months (95%CI: 1.4–7.8)	6.9 months (95%CI: 2.46–NE)	Breathlessness (42%), fatigue (36%), decreased appetite (30%), oral mucositis (24%), and nausea (24%)	Lorimer et al^418^
Dabrafenib + trametinib	Retrospective study	27 (9 treated with dabrafenib and trametinib)	33.3	66.7	0	0	270 days	475 days	Recurrent fever (11%), fever and hypertension (33%), fever (22%), hypertension (22%)	Tiago et al^419^
Dabrafenib + trametinib	Retrospective study	5	20	40	20	20	NA	NA	Anorexia (80%), nausea (60%), fatigue (40%), hepatotoxicity (40%), and upper gastrointestinal bleeding (20%)	Fernanda et al^420^
Dabrafenib + trametinib	Phase 2 clinical trial	16	63	6	19	13	6.7 months (95%CI: 4.7–13.8)	14.5 months (95%CI: 6.8–23.2)	Fatigue (38%), pyrexia (37%), and nausea (35%)	Vivek et al^421^^,^^422^
Sorafenib	Phase 2 clinical trial	20	10	NA	25	10	1.9 months (95%CI: 1.3–3.6)	3.9 months (95%CI: 2.2–7.1)	Rash/desquamation (65%), fatigue (60%), hypoglycemia (30%), mucositis (35%), and nausea (25%)	Panayioti et al^423^
Sorafenib	Phase 2 clinical trial	10	0	0	40	0	2.8 months (95%CI: 0.7–5.6)	5.0 months (95%CI: 0.7–5.7)	Palmar-plantar erythrodysesthesia (50%), alopecia (30%), hypertension (50%), and diarrhea (30%)	Yasuhiro et al^424^
Vemurafenib	Phase 2 clinical trial	7	14	14	0	57	NA	NA	NA	David et al^425^

Note: CI, confidence interval; CR, complete response; mOS, median overall survival; mPFS, median progression-free survival; NA, not accessed; NE, not estimable; PD, progression disease; PR, partial response.

**Table 4:** Case reports about dabrafenib in treating anaplastic thyroid cancer.

Reference	Age (years)	Gender	Stage	CTR	RTR	Surgery	Usage of dabrafenib	Outcome
Rishi et al^426^	47	Female	IVB	CPL, PTX, CDDP, DOX	+	+	Dabrafenib (oral, 150 mg twice daily) + trametinib (2 mg daily)	9-month clinical and radiologic response of metastatic lung nodules
Annette et al^427^	49	Female	IVB	PTX	+	–	Dabrafenib (150 mg twice daily)	3-month metabolic response in all sites of disease and a radiologically RECIST partial response
Annette et al^427^	67	Male	IVB	–	+	–	Dabrafenib (150 mg twice daily)	11-week reduction in tumor-related symptoms and size of palpable thyroid mass
Jennifer et al^428^^;^^429^	60	Male	IVB	PTX + CPL	+	+	Dabrafenib (oral, 150 mg twice daily) + trametinib (2 mg daily) + pembrolizumab (200 mg, IV)	Pre-operative: a significant partial response, and the tumor was deemed resectable Post-operative: disappeared nodules, and excellent quality of life
Jennifer et al^428^	48	Female	IVB	PTX ± CPL	+	+	Dabrafenib (oral, 150 mg twice daily) + trametinib (oral, 2 mg daily)	Pre-operative: 1-month >50% tumor reduction, improved dysphagia and dyspnea Post-operative: no evidence of disease progression for one year until bone metastases
Jennifer et al^428^	69	Female	IVB	PTX ± CPL	+	+	Dabrafenib (oral, 150 mg twice daily) + trametinib (oral, 2 mg daily)	Pre-operative: resolved dyspnea and resume full oral diet Post-operative: significant reduction of the primary tumor and lymphadenopathy as well as separation from the carotid
Jennifer et al^428^	58	Male	IVB	PTX ± CPL	+	+	Dabrafenib (oral, 150 mg twice daily) + trametinib (oral, 2 mg daily) + pembrolizumab (200 mg, every 3 weeks)	Resolution of dysphagia and dyspnea, resumption of an oral diet, and no disease progression (at least 20 months from diagnosis)
Jennifer et al^428^	73	Female	IVC	–	–	+	Dabrafenib (oral, 150 mg twice daily) + trametinib (oral, 2 mg daily) + pembrolizumab (2 mg/kg, IV)	Pre-operative: near-complete metabolic response Post-operative: no evidence of disease progression
Jennifer et al^428^	46	Female	IVC	PTX ± CPL	+	+	Dabrafenib (150 mg twice daily, oral) + trametinib (oral, 2 mg daily) + pembrolizumab (200 mg, every 3 weeks)	Pre-operative: marked reduction of primary disease and resolution of metabolically active systemic disease Post-operative: no disease progression (12 months from diagnosis)
Maria et al^430^	74	Female	IVC	PTX	–	+	Dabrafenib + trametinib + pembrolizumab	Pre-operative: 11-month response (a decrease in the size of primary tumor, an almost complete metabolic response, and improvement in distant metastatic disease) Post-operative: 9-month no evidence of progression
Hilary et al^431^	73	Male	NA	+	+	+	Dabrafenib (150 mg twice daily) + trametinib (2 mg daily)	Pre-operative: strong partial response (marked reduction in the size of the thyroid mass and cervical adenopathy) Post-operative: NA
Johnathan et al^432^	67	Male	NA	PTX, 5-ﬂuorouracil, and hydroxyurea	+	+	Dabrafenib + trametinib	Pre-operative: decreased tumor size Post-operative: 17-month no evidence of disease
Lin et al^433^	61	Female	NA	–	–	+	Anlotinib (10 mg, once a day, 2 weeks on/1 week off) + sintilimab (200 mg, every 3 weeks) + dabrafenib (75 mg, twice daily) + trametinib (2 mg, daily)	Pre-operative: pathological complete response Post-operative: remission with an excellent quality of life
Elisabeth et al^434^	73	Male	IVB	–	–	+	Pre-operative: lenvatinib (20 mg, daily) + dabrafenib (150 mg, twice daily) + trametinib (4 mg, daily) Post-operative: lenvatinib and pembrolizumab after local recurrence	Pre-operative: residual local tumor without lymphadenopathy, decreased initial tumor infiltration, and extensive regressive necrosis in the primary tumor Post-operative: 8-month stable disease
Yuntao et al^435^	65	Female	IVC	–	–	+	Pre-operative: dabrafenib (oral, 150 mg twice daily) + trametinib (oral, 2 mg daily) + sintilimab (IV, 200 mg, every 3 weeks) Post-operative: sintilimab (IV, 200 mg, every 3 weeks)	Pre-operative: shrink of primary tumor, separation from the carotid, and nearly disappeared metastatic lung disease Post-operative: 12-month no evidence of disease

Note: CTR, chemotherapy; CPL, carboplatin; CDDP, cisplatin; DOX, doxorubicin; IV, administered intravenously; NA, not available; PTX, paclitaxel; RECIST, response evaluation criteria in solid tumors; RTR, radiotherapy.

**Table 5:** Efficacy and safety results from clinical studies of immunotherapies for anaplastic thyroid cancer.

Treatment	Design	Cases	PR (%)	CR (%)	SD (%)	PD (%)	mPFS	mOS	Adverse events	Reference
Pembrolizumab + docetaxel/DOX + RTR	Phase 2 clinical trial	3	0	0	0	100	NA	2.76 months	Pneumonitis (67%), respiratory failure (67%), laryngeal edema (33%), and lung infection (33%)	Ashish et al^436^
Spartalizumab	Phase 2 clinical trial	42	12	7	0	0	1.7 months (95%CI: 1.2–1.9)	5.9 months (95%CI: 2.4–NE)	Diarrhea (12%), pruritus (12%), fatigue (7%), and pyrexia (7%)	Jaume et al^437^
Tremelimumab + durvalumab + RTR	Pilot study	12	0	0	8.3	NA	NA	104 days (range: 12–622)	Fatigue (83%), cough (75%), dysphagia (67%), constipation (58%), edema limbs (58%), and oral pain (58%)	Nancy et al^438^
Pembrolizumab + lenvatinib/trametinib/dabrafenib and trametinib	Retrospective study	12	42	0	33	25	6.93 months (95%CI: 1.7–12.15)	2.96 months (95%CI: 2.2–3.7)	Fatigue (91.7%), anemia (83.3%) hypertension (66.7%), and dry mouth (66.7%)	Priyanka et al^439^
Lenvatinib + pembrolizumab	Retrospective study	6	66	0	16	16	16.8 months	17.3 months	Hypertension (83%), anorexia (33%), diarrhea (33%), fatigue (33%), and proteinuria (33%)	Christine et al^440^
pembrolizumab/nivolumab	Retrospective study	13	15	NA	23	NA	1.9 months (IQR: 9.0)	3.9 months (IQR: 15.7)	Endocrinopathies (23%), rash (15%), nervous disorder (15%), and musculoskeletal disorder (8%)	Alycia et al^441^

Note: CI, confidence interval; CR, complete response; DOX, doxorubicin; IQR, interquartile range; mOS, median overall survival; mPFS, median progression-free survival; NA, not accessed; NE, not estimable; PD, progression disease; PR, partial response; RTR, radiotherapy.

**Table 6:** Case reports about immunotherapies for anaplastic thyroid cancer.

Reference	Age (years)	Gender	Stage	CTR	RTR	Surgery	Immunotherapy	Effect
Elisabeth et al^434^	57	Male	IVC	CPL, PTX	–	+	Lenvatinib (20 mg, daily) + pembrolizumab (200 mg)	Pre-operative: regression in size and disappearance of the small bilobular lung metastases Post-operative: 11-month stable disease
Revathi et al^442^	62	Male	NA	DOX, CDDP, PTX	–	+	Vemurafenib + nivolumab	Vemurafenib only: mixed response Vemurafenib + nivolumab: 20-month complete radiographic and clinical remission
Marra Jai et al^443^	53	Male	IVC	DOX	+	–	Pembrolizumab (200 mg)	Reduction in total tumor burden
Ammar et al^444^	49	Male	NA	DOX	+	+	Pembrolizumab	Diffuse bone metastasis and a new liver lesion
Ammar et al^444^	61	Female	IVC	DOX, CPL, PTX	+	+	Dabrafenib (oral, 150 mg, twice daily) + trametinib (oral, 2 mg, daily) + pembrolizumab	Shrinkage in the size of lung metastases and 10-month stable disease
Luming et al^445^	55	Female	IVB	–	+	+	Apatinib (250 mg, daily) + camrelizumab	Local recurrence, but the 11-month clinical stable stage
Lin et al^446^	67	Female	IVB	–	–	+	Sintilimab (200 mg, every 3 weeks) + anlotinib (oral, 12 mg, once daily, 2-week on/1-week off)	RECIST partial response and an excellent quality of life
Shyang-Rong et al^447^	58	Male	IVC	DOX	+	+	Pembrolizumab (200 mg, every three weeks) + lenvatinib (20 mg/day and 10 mg/day alternatively)	Transient decrease of pulmonary nodules and new spinal metastases
Shyang-Rong et al^447^	71	Male	IVC	–	–	–	Pembrolizumab (50 mg, twice) + lenvatinib (oral, 20 mg/day and 10 mg/day alternatively)	Neck tumor and lymphadenopathies and multiple enlarged pulmonary metastases
Shyang-Rong et al^447^	59	Male	IVB	DOX	–	–	Spartalizumab (400 mg, every 4 weeks)	Unchanged size of neck mass remained, and enlargement after 23-month treatment
Shyang-Rong et al^447^	60	Female	IVC	–	+	+	i) Sorafenib (400 mg once daily for 2 weeks + 600 mg once daily for another week); ii) pembrolizumab (200 mg) + lenvatinib (10 mg once daily, finally 24 mg once daily); iii) dabrafenib (oral, 300 mg daily) + trametinib (oral, 2 mg daily)	Sorafenib: enlargement of neck mass Pembrolizumab + Lenvatinib: shrinkage of neck and pulmonary nodules Dabrafenib + Trametinib: neck tumor progressed after 10.2-month treatment
Maxwell et al^448^	60s	Female	NA	CPL, PTX	+	+	i) Nivolumab; ii) dabrafenib + trametinib + nivolumab; iii) FS118 (20 mg/kg weekly)	Nivolumab: partial response Dabrafenib + trametinib + nivolumab: 1-month fever, drug-induced liver injury FS118: sustained RECIST partial response
Doreen et al^449^	51	Female	IVC	–	+	+	Pembrolizumab	Complete RECIST tumor regression of both primary and lung metastasis
Yurou et al^450^	47	Female	NA	–	+	+	Tislelizumab (200 mg, every three weeks)	Partial response and no tumor recurrence

Note: CTR, chemotherapy; CPL, carboplatin; CDDP, cisplatin; DOX, doxorubicin; NA, not available; PTX, paclitaxel; RECIST, response evaluation criteria in solid tumors; RTR, radiotherapy.

**Table 7:** Ongoing clinical trials of agents for anaplastic thyroid cancer.

Title	NCT number	Starting date	Status	Enrolment	Interventions	Organization	Country
interNational Anaplastic Thyroid Cancer Tissue Bank and Database (iNATT) (iNATT)	NCT01774279	2013–06	Recruiting	350	Tissue, blood, and clinical data collection	Velindre NHS Trust	United Kingdom
Trametinib in Combination with Paclitaxel in the Treatment of Anaplastic Thyroid Cancer	NCT03085056	2017-03-15	Active, not recruiting	13	Trametinib, paclitaxel	Memorial Sloan Kettering Cancer Center	United States
Atezolizumab With Chemotherapy in Treating Patients with Anaplastic or Poorly Differentiated Thyroid Cancer	NCT03181100	2017-07-27	Active, not recruiting	50	Atezolizumab, bevacizumab, cobimetinib, nab-paclitaxel, paclitaxel, vemurafenib	M.D. Anderson Cancer Center	United States
Nivolumab Plus Lenvatinib Against Anaplastic Thyroid Cancer (NAVIGATION)	NCT05696548	2019-07-02	Active, not recruiting	51	Lenvatinib, nivolumab	National Cancer Center Hospital East	Japan
Study of Cemiplimab Combined with Dabrafenib and Trametinib in People with Anaplastic Thyroid Cancer	NCT04238624	2020-01-20	Recruiting	15	Dabrafenib, trametinib	Memorial Sloan Kettering Cancer Center	United States
Abemaciclib in Metastatic or Locally Advanced Anaplastic/Undifferentiated Thyroid Cancer	NCT04552769	2020-09-10	Active, not recruiting	9	Abemaciclib	Stanford University	United States
Dabrafenib, Trametinib, and IMRT in Treating Patients with BRAF Mutated Anaplastic Thyroid Cancer	NCT03975231	2020-09-14	Recruiting	6	Dabrafenib, trametinib, and intensity-modulated radiation therapy	City of Hope Medical Center	United States
Pembrolizumab, Dabrafenib, and Trametinib Before Surgery for the Treatment of BRAF-Mutated Anaplastic Thyroid Cancer	NCT04675710	2021-06-24	Recruiting	30	Dabrafenib, trametinib, pembrolizumab, conventional surgery, intensity-modulated radiation therapy, and quality-of-life assessment	M.D. Anderson Cancer Center	United States
Lenvatinib and Pembrolizumab for the Treatment of Stage IVB Locally Advanced and Unresectable or Stage IVC Metastatic Anaplastic Thyroid Cancer	NCT04171622	2021-11-04	Recruiting	25	Lenvatinib, pembrolizumab	M.D. Anderson Cancer Center	United States
The Efficacy and Safety of HLX208 in Advanced Anaplastic Thyroid Cancer (ATC) With BRAF V600 Mutation	NCT05102292	2021-12-10	Active, not recruiting	25	HLX208	Shanghai Henlius Biotech	China
IMRT Followed by Pembrolizumab in the Adjuvant Setting in Anaplastic Cancer of the Thyroid (IMPAACT): Phase II Trial Adjuvant Pembrolizumab After IMRT in ATC	NCT05059470	2022-02-11	Recruiting	35	Pembrolizumab	M.D. Anderson Cancer Center	United States
Phase II Trial of Pembrolizumab in Metastatic or Locally Advanced Anaplastic/Undifferentiated Thyroid Cancer	NCT05119296	2022-02-15	Recruiting	20	Pembrolizumab (Keytruda)	Stanford University	United States
Vudalimab for the Treatment of Locally Advanced or Metastatic Anaplastic Thyroid Cancer or Hurthle Cell Thyroid Cancer	NCT05453799	2022-07-21	Recruiting	54	Vudalimab	Northwestern University	United States
PD-1 Inhibitor and Anlotinib Combined with Multimodal Radiotherapy in Recurrent or Metastatic Anaplastic Thyroid Cancer	NCT05659186	2022-12-30	Recruiting	20	Tislelizumab, anlotinib, and radiotherapy	West China Hospital	China
NEO- and Adjuvant Targeted Therapy in Braf-mutated Anaplastic Cancer of the Thyroid (NEO-ATACT Study) (NEO-ATACT)	NCT06079333	2023-01-01	Recruiting	20	Dabrafenib/trametinib	Leiden University Medical Center	Netherlands
Efficacy of Pembrolizumab and Lenvatinib in Patients with Anaplastic Thyroid Cancer	NCT06374602	2024-03-25	Recruiting	20	Pembrolizumab + lenvatinib	Saint Petersburg State University	Russia
Study of the Rechallenge Concept in Patients With BRAF-positive Anaplastic Thyroid Cancer After Progression on Anti-BRAF Therapy	NCT06362694	2024-03-25	Recruiting	34	Dabrafenib + trametinib	Saint Petersburg State University	Russia
Efficacy of Pembrolizumab and Lenvatinib in Patients with Anaplastic Thyroid Cancer	NCT06374602	2024-03-25	Recruiting	20	Pembrolizumab + lenvatinib	Saint Petersburg State University	Russia

Note: All data in this table was collected from the public information uploaded on ClinicalTrials.gov. NCT, National Clinical Trial; NHS, National Health Service.

### Targeting MAPK signaling pathway

#### Targeting RTKs

##### Anlotinib

As a VEGFR2 inhibitor, anlotinib showed an anti-tumor effect *in vitro*. Anlotinib blocked the angiogenesis in ATC by targeting the CXCL11-EGF-EGFR positive feedback loop.^187^ Autophagic blockade enhanced anlotinib-mediated ferroptosis in ATC.^188^ A cohort of ATC patients receiving anlotinib-based chemotherapy (*n* = 25) had 25.1-week median progression-free survival and 96.0-week median disease specification survival, and the objective remission rate and disease control rate were 60% and 88%, respectively.^189^

##### Apatinib

Apatinib, a VEGFR2 inhibitor, induced both autophagy and apoptosis by inhibiting AKT-mTOR pathway.^190^ It led a 93-year-old female ATC patient to have stable disease with a best response of 19.7% of the primary lesion, sustained shrinkage of tumor and metastatic lymph node, and 41-week OS.^191^ The combination of ^125^I and apatinib caused a significant reduction in tumor size in 49-year-old female ATC patients.^192^ In a phase II trial of apatinib (*n* = 17), the disease control rate was 88.2%, but treatment was terminated in 23.5% of patients due to intolerable toxicity.^193^ Interestingly, the combination of apatinib and melittin showed an extra anti-tumor effect via caspase-1-GSDMD and caspase-3-GSDME pyroptosis.^193^

##### Lenvatinib

Lenvatinib, a multi-tyrosine kinase inhibitor targeting VEGFRs, PDGFRs, and FGFR1, demonstrated significant anti-proliferative effects in ATC. It exhibited promising inhibitory actions on tumor growth^194^ and brain metastasis,^195^ particularly by impeding angiogenesis. Notably, lenvatinib displayed inhibitory effects on BRAF^WT/V600E^-ATC cells, especially in the presence of pericytes enriched in ATC samples.^196^ However, no significant correlation was observed between VEGFR2 expression in tumor tissue and clinical response to lenvatinib among ATC patients.^197^

The development of a lenvatinib-loaded nanocomposite showed therapeutic potential *in vivo*.^198^ Combination therapies involving lenvatinib with other agents like DOX,^199^ HNHA (a histone deacetylase inhibitor),^200^ IRAK1/4 Inhibitor I,^201^ MEK inhibitors,^202^ PTX,^203^ and vinorelbine^204^ demonstrated synergistic effects, surpassing the individual agents' impact. Combinations with anti-PD-1/PD-L1 therapy revealed a reduction in polymorphonuclear myeloid-derived suppressor cells while combining lenvatinib with anti-Gr-1 antibody showed an expanded myeloid-derived suppressor cell population along with enhanced anti-tumor effects compared with lenvatinib monotherapy.^205^

Clinical studies involving lenvatinib in ATC are detailed in Table 2, showcasing its promising potential in specific cases. Notably, a 68-year-old IVB ATC male patient experienced a 21-month survival post-trimodal treatment,^206^ and a 54-year-old woman with paucicellular metastatic ATC showed an 18-month partial tumor response in lung metastasis after receiving a lenvatinib–pembrolizumab combination.^207^

**Table 2:** Efficacy and safety results from clinical studies of lenvatinib in anaplastic thyroid cancer.

Design	Cases	PR (%)	CR (%)	SD (%)	PD (%)	mPFS	mOS	Adverse events	Reference
Phase 2 clinical trial	17	24	0	71	6	7.4 months (95%CI: 1.7–12.9)	10.6 months (95%CI: 3.8–19.8)	Decreased appetite (82%), hypertension (82%), fatigue (59%), nausea (59%), and proteinuria (59%)	Makoto et al^407^
Phase 2 clinical trial	34	2.9	0	0	0	2.6 months (95%CI: 1.4–2.8)	3.2 months (95%CI: 2.8–8.2)	Hypertension (56%), decreased appetite (29%), fatigue (29%), and stomatitis (29%)	Lori et al^408^
Phase 2 clinical trial	52	9.5	2.4	61.9	21.4	NA	5.0 months (95%CI: 2.7–6.9)	Loss of appetite (48.0%), fatigue (48.0%), hypertension (44.0%), and palmar-plantar erythrodysesthesia syndrome (26.0%)	Takuya et al^409^
Pilot study	12	33	NA	25	NA	i) No FGFR4 intensity: 0.5 months; ii) weak FGFR4 intensity: 3.2 months (95%CI: 1.1–NE); iii) moderate FGFR4 intensity: 4.6 months (95%CI 1.1–NE)	NA	NA	Haruhiko et al^410^
Retrospective study	23	17.3	NA	26.1	30.4	NA	166 days	Hypertension (91%), general fatigue and anorexia (65%), proteinuria (61%), and tumor-skin fistulas (26%)	Hiroyuki et al^411^
Retrospective study	5	60	0	40	0	NA	165 days	Hypertension (80%), diarrhea (40%), fatigue (80%), and decreased appetite (80%)	Satoshi et al^412^
Retrospective study	18	NA	NA	NA	NA	NA	230 days (range: 64–839)	NA	Soo Young et al^413^
Retrospective study	14	29	0	64	7	5.7 months (95%CI: 2.2–8.3)	6.7 months (95%CI: 3.0–8.4)	Hypertension (86%), loss of appetite (86%), fatigue or asthenia (79%), proteinuria (79%), and hypothyroidism (79%)	Mijin et al^414^
Retrospective study	56 (36 treated with lenvatinib)	33.3	NA	52.8	11.1	3.5 months (95%CI: 2.3–5.37)	4.77 months (95%CI: 3.07–6.50)	Hypertension (80.6%), loss of appetite (50.0%), cavitation (47.2%), proteinuria and fatigue (41.7%), necrosis (38.9%), cutaneous fistula (33.3%), and tracheal fistula (25.0%)	Hiroyuki et al^415^
Retrospective study	16 (10 treated with lenvatinib)	38	NA	38	12	2.6 months (95%CI: 1.8–NE)	3.9 months (95%CI: 2.5–NE)	Hypertension (70%), pain (70%), fatigue (70%), mucositis (50%), and hand–foot skin reaction (30%)	Priyanka et al^416^
Observational study	124	41	2.9	32.4	23.8	NA	101.0 days (95%CI: 80.0–130.0)	Hypertension (70.2%), proteinuria (29.8%), palmar-plantar erythrodysesthesia syndrome (25.8%), and hematological toxicity (33.9%)	Shunji et al^417^

Note: CI, confidence interval; CR, complete response; FGFR4, fibroblast growth factor receptor 4; mOS, median overall survival; mPFS, median progression-free survival; NA, not accessed; NE, not estimable; PD, progression disease; PR, partial response.

Despite its promise, the safety and efficacy of lenvatinib are under scrutiny. Apart from the adverse events listed in Table 2, instances like posterior reversible encephalopathy syndrome in a 66-year-old female ATC patient^208^ and bilateral pneumothorax in an ATC patient with lung metastasis during lenvatinib therapy have been reported.^209^ Furthermore, the partial response defined in Response Evaluation Criteria in Solid Tumors (RECIST) remains elusive in some cases after successful local control of metastatic ATC (*n* = 3).^210^ Notably, a meta-analysis highlighted common adverse events such as hypertension (56.6%), proteinuria (32.6%), and fatigue (32%).^211^ Addressing the safety and efficacy concerns of lenvatinib will be imperative in future studies.

##### Pazopanib

Pazopanib targets several RTKs (VEGFRs, PDGFRβ, and FGFR1). It inhibited the proliferation of primary human ATC cells,^212^ and the combination of pazopanib and other agents (like PTX^213^ or topotecan^214^) showed more synergistically anti-tumor effect. However, results from clinical trials of pazopanib in ATC were disappointing. In a phase 2 trial of pazopanib in ATC (*n* = 15), there were no confirmed RECIST responses, and treatment was discontinued because of severe adverse events.^215^ In another phase 2 trial (*n* = 71), there was no difference in mOS between the pazopanib group (5.7 months; 95%CI: 4.0–12.8 months) and placebo group (7.3 months; 95%CI: 4.3–10.6 months) (HR = 0.86; 95%CI: 0.52–1.43; one-sided log-rank *P* = 0.28).^216^

##### Sorafenib

As a multi-target inhibitor, sorafenib blocks not only RTKs (VEGFRs and PDGFR) but also RAF-1. Sorafenib blocked the proliferation of vascular endothelial cells stimulated by ATC cells.^217^ The combinations of sorafenib and other agents have been examined in ATC. A combination of sorafenib and metformin^218^ or HNHA^219^ showed a synergistically anti-proliferative effect on ATC cells and cancer stem cells. Sorafenib can also synergize with other drugs (adavosertib,^220^ centrinone,^221^ quinacrine,^222^ and withaferin A^223^) in blocking the tumor growth of ATC. Besides, the sorafenib-radiation-HNHA^224^ or sorafenib-radiation-PTX^225^ therapy showed therapeutic potential on ATC *in vivo*. Clinical studies of sorafenib in ATC are listed in Table 3.

##### Sunitinib

Sunitinib blocked VEGFR2 and PDGFRβ, but it exhibited no effect on the proliferation of ATC cells.^217^ Combination of sunitinib and irinotecan showed synergistic anti-tumor activity on ATC *in vitro* and *in vivo*.^226^ Clinical applications of sunitinib in treating ATC were limited. The combination of radiotherapy, chemotherapy, and sunitinib led a 49-year-old female ATC patient to have a complete response and remains without evidence of disease more than 18 months after diagnosis,^227^ and it also caused a reduction in tumor size and complete macroscopic response in a 79-year-old male ATC patient unfit for systemic chemotherapy treatment.^228^

##### Vandetanib

As an effective inhibitor of VEGFR2/3 and EGFR, vandetanib inhibited angiogenesis and development of ATC *in vivo* and *in vitro*.^229^^,^^230^ Both lenvatinib and vandetanib blocked the proliferation and promoted apoptosis in primary ATC cells,^231^ and vandetanib showed a more inhibitory effect on ATC cell proliferation and angiogenesis than sorafenib *in vivo*.^232^ However, more clinical trials of vandetanib are necessary to check its efficacy and safety.

#### Targeting RAF (BRAF, BRAF^V600E^)

##### Dabrafenib

Dabrafenib demonstrated effective inhibition of CRAF and BRAF^V600E^, inducing G0/G1-arrest by reducing MEK/ERK phosphorylation, presenting a promising avenue for ATC treatment.^233^ Studies have unveiled the synergistic potential of dabrafenib in combination therapies for ATC. Notably, the combined therapy of dabrafenib with trametinib (MEK inhibitor) is recommended, especially for managing stage IVB/IVC BRAF^V600E^-positive ATC.^175^ Additionally, *in vitro* observations indicate the anti-tumor effects of dabrafenib in conjunction with other agents such as axitinib,^234^ epigallocatechin-3-gallate,^235^ erlotinib,^236^ and melatonin.^237^ However, further independent replications are essential to validate the safety and efficacy of these experimental combination therapies. Clinical studies and case reports of dabrafenib in ATC are detailed in Tables 3 and 4, respectively. Despite its potential, the efficacy of dabrafenib requires enhancement due to several confining factors affecting its visceral distribution, including drug lipophilicity, rapid target dissociation, and high albumin binding.^238^ Moreover, the emergence of RAC1^P34R^ mutation has been linked to dabrafenib resistance in the anaplastic transformation of PTC.^239^ Strategies targeting the reactivated RAS signaling pathway, such as SHP099 (a SHP2 inhibitor), have shown potential in reversing resistance to dabrafenib in ATC.^240^ However, a comprehensive exploration into the mechanisms of resistance to dabrafenib and related solutions is yet to be undertaken.

##### PLX4720

PLX4720, a specific BRAF^V600E^ inhibitor, restrained the development of ATC *in vivo*.^241^ The same effect was observed *in vivo* when combined with thyroidectomy.^242^ Combination of PLX4720 and anti-PD-1/PD-L1 antibody improved the survival of the murine ATC model,^243^ and the combination of PLX4720 and oncolytic herpes simplex virus enhanced the anti-tumor effect with PD-1 blockade.^244^ More clinical studies are suggested for the potential therapeutic effect of PLX4720.

##### Vemurafenib

Vemurafenib, acting as a specific inhibitor of BRAF^V600E^, exhibited promising effects on ATC *in vitro*.^245^ It enhanced TRAIL-induced apoptosis and, intriguingly, promoted self-renewal in ATC cells by activating the sonic hedgehog pathway.^246^ Moreover, *in vitro* studies demonstrated that vemurafenib increased the cytotoxicity of apigenin^247^ and tunicamycin^248^ on ATC and displayed synergistic therapeutic effects when combined with metformin.^249^ Notably, the inhibition of STAT3 demonstrated a reduction in resistance to vemurafenib in ATC.^250^ A clinical study of vemurafenib in ATC is outlined in Table 3, where it exhibited rapid improvements in the condition of ATC patients, significantly supporting subsequent radiation therapy.^251^ Case reports also indicated that vemurafenib improved symptoms in patients with specific mutations, such as BRAF^V600E^ and mutant-TP53 (c.550G > A, p.E180K).^252^ However, outcomes varied, as seen in the case of a 51-year-old male ATC patient who initially responded to vemurafenib but experienced rapid clinical deterioration.^253^ Further clinical investigations of vemurafenib in ATC are warranted to comprehensively evaluate its efficacy and safety in this context.

##### Other potential targeting

While targeting RTK and RAS remains a focus in ATC research, investigations targeting other components within the MAPK signaling pathway are gaining importance and warrant further attention. The prospect of targeting RAS is promising for future therapeutic approaches. Combined treatment involving salirasib (a RAS inhibitor) and modified citrus pectin demonstrated therapeutic effects in ATC.^254^ Similarly, sulforaphane enhanced the efficacy of photodynamic therapy in ATC by specifically targeting the MAPK pathway.^255^ Expanding beyond trametinib, alternate strategies for MEK targeting in ATC are being explored. For instance, the combination of PLX51107 (a BET inhibitor) and PD0325901 (a MEK inhibitor) showcased therapeutic potential in ATC by targeting MYC transcription.^256^ Intriguingly, while dual inhibition of BRAF^V600E^ and MEK failed to impede SW1736 ATC cell migration in 2D culture, it significantly reduced SW1736 cell invasion in 3D culture settings.^257^ Studies focusing on targeting ERK in ATC are scarce. Inhibition of ERK dimerization emerged as a strategy to suppress ERK activation, ultimately impeding the proliferation and metastasis of BRAF-mutant ATC.^258^ Additionally, epigallocatechin-3-gallate exhibited inhibitory effects on ATC cell proliferation and induced apoptosis by targeting the EGFR-ERK pathway and the cyclin B1-CDK1 complex.^259^

### Targeting PI3K-AKT-mTOR signaling pathway

#### Targeting PI3K

Blocking PI3K in ATC was rarely examined. The cytotoxicity of two heat-shock protein 90 (HSP90) inhibitors, 17-AAG and herbimycin A, is associated with the suppression of PI3K-AKT signaling in ATC.^260^ Nanoparticles loaded 17-AAG and Torin2 blocked ATC cell growth and improved mOS of murine ATC models by targeting VEGFR2.^261^ Dual inhibition of PI3K and PLK1 also induced apoptosis and suppressed tumor growth of ATC significantly.^262^ Metformin inhibited the PI3K-AKT-FOXO1 pathway in SW1736 and 8305C ATC cell lines but failed to regulate AKT in the C643 cell line and phosphorylation status of PI3K, AKT, and FOXO1 in all three ATC cell lines.^263^ Combination of metformin and pioglitazone blocks PI3K-Akt-mTOR pathway and up-regulates several tumor suppressor genes (including PTEN) in SW1736 and C643 ATC cell lines.^264^

#### Targeting AKT

Targeting AKT in ATC has been gradually tested *in vitro* and *in vivo*. A combination of MK-2206 (AKT inhibitor) and tyrphostin AG 1296 (PDGFR inhibitor) inhibited the tumor growth of ATC synergistically.^265^ Combination of baicalein and docetaxel significantly suppressed proliferation and induced apoptosis by down-regulating apoptotic and angiogenic protein expression and blocking ERK and Akt/mTOR pathways in ATC.^266^ High iodine promoted ATC cell proliferation via AKT-mediated Wee1/CDK1 axis,^267^ and diallyl trisulphide compromised the phenotype of ATC cancer stem cells and restored thyroid-specific gene expression of ATC cells by targeting AKT-SOX2 pathway.^268^ Both berberine^269^ and salmonella^270^ activated autophagy and inhibited ATC tumor growth by blocking the AKT-mTOR pathway. A self-assemble peptide drug inhibited AKT1 at the half maximal inhibitory concentration (IC_50_) of 18.2 μM and 12.4 μM in 8305C and 8505C ATC cell lines, respectively.^271^

#### Targeting mTOR

The mTOR pathway has emerged as a potential therapeutic target in ATC.^272^ Everolimus, an mTOR inhibitor, demonstrated promising effects in ATC *in vitro*.^273^ A phase II study involving everolimus, encompassing 33 participants (including 7 ATC cases), revealed two patients with partial response and stable disease for 17.9 and 26 months, respectively.^274^ Notably, one patient exhibited a partial response for 27.9 months, while two others had stable disease for 3.7 and 5.9 months, respectively.^275^ Combining BP-14 (a CDK inhibitor) with everolimus revealed a robust synergistic effect in inhibiting the proliferation of FRO, SW1736, and 8505C ATC cell lines.^276^ Nevertheless, the presence of a nonsense mutation in TSC2 (TSC2^Q1178^^∗^) enhanced sensitivity to everolimus, while an mTOR mutation (mTOR^F2108L^) conferred resistance.^277^ Comprehensive evaluations focusing on both efficacy and safety are imperative to validate the use of everolimus in ATC treatment. Apart from everolimus, alternative agents targeting mTOR are being explored. The combination of AZD6244 (a MEK inhibitor) and rapamycin demonstrated superior growth inhibition compared with individual agents across 10 DTC and ATC cell lines.^278^ Vistusertib effectively overcame resistance to PTX and suppressed ATC tumor growth.^279^ Additionally, the paeonol-platinum (II) complex exhibited cytotoxic effects on the SW1736 ATC cell line by down-regulating the mTOR pathway,^280^ while monensin hindered ATC cell proliferation by impeding mitochondrial function and AMPK-mTOR signaling.^281^

#### Other hopeful targeting therapies

Some other targeted therapies have been explored in the relentless pursuit of effective treatments for ATC. Here we summarized other ATC therapies targeting ALK, CDKs, histone deacetylases, TERT, and TP53.

#### Targeting ALK

It was first reported that targeting ALK by crizotinib (an ALK inhibitor) showed an excellent response in an ATC case harboring ALK rearrangement.^282^ Four years later, this case was found to develop secondary resistance to crizotinib, and administering two ALK inhibitors (ceritinib and brigatinib) brought a therapeutic response to the patient.^283^ Although the patient died of locally advanced squamous esophageal cancer induced by radiotherapy, targeting ALK rearrangements is still hopeful in future ATC treatment.

#### Targeting CDKs

Recognizing the frequent inactivation of negative cell cycle regulators and copy number gains of cyclins in ATC, cell cycle inhibitors have emerged as potential therapeutic candidates. CDK7 was associated with poor clinical prognosis of ATC, and one of its covalent inhibitors, THZ1, was identified by high-throughput chemical screening and evaluated to be effective in inhibiting the activity of cancer stem cells in ATC.^284^^,^^285^ THZ531 (a covalent inhibitor of CDK12 and CDK13) induced cell cycle arrest and apoptosis by blocking CDK12 *in vitro*.^286^ Two CDK4/6 inhibitors, ribociclib and abemaciclib, induced cell cycle arrest and apoptosis in ATC.^287^^,^^288^ Another CDK4/6 inhibitor, palbociclib, induced cell cycle arrest in the G_0_/G_1_ phase only in ATC cell lines with CDKN2A/CDKN2B mutation rather than those with wide-type alternatives.^289^ Two broad-spectrum inhibitors of CDKs, dinaciclib and flavopiridol, showed tumor-suppressing effects on ATC *in vitro* and *in vivo*.^290^^,^^291^ In the future, new strategies for treating ATC using CDK inhibitors will be available.

### Targeting histone deacetylases

Histone deacetylase inhibitors have been gradually explored in ATC treatment. They blocked ATC cell migration and invasion by inducing the expression of E-cadherin and proper membrane localization of E-cadherin/β-catenin complex,^292^ and improved radioiodine effect in PDTC and ATC by regulating the expression of NIS, thyroid peroxidase, and thyroglobulin.^293^ Here we summarized three histone deacetylase inhibitors tested in ATC.

#### Belinostat

Belinostat reduced tumor size in a xenograft model of ATC.^294^ It had a synergistic activity with HSP90 inhibitor NVP-AUY922 in causing cytotoxicity.^295^ Interestingly, several histone deacetylase inhibitors (belinostat, vorinostat, and trichostatin A) synergized with HSP90 inhibitor SNX5422 in inducing cytotoxicity,^296^ while both sodium butyrate and trichostatin A induced apoptosis and differential cell cycle arrest *in vitro*.^297^

#### Panobinostat

Panobinostat induced radioiodine by up-regulating NIS,^298^ and significant tumor reduction induced by panobinostat was observed.^299^ Compared with sorafenib and selumetinib, panobinostat showed maximum cytotoxicity in patient-derived tumor tissue of ATCs/PDTCs at the minimum dosage.^300^

#### Valproic acid

Exploration of valproic acid (VPA) in combination with various agents for ATC warrants heightened attention. *In vitro* studies revealed VPA's augmentation of DOX and PTX effects.^301^^,^^302^ Compared with sole imatinib treatment, the combination of VPA with imatinib demonstrated more pronounced cell cycle arrest.^303^ VPA prompted apoptosis in the KAT-18 ATC cell line, showing similar effects when combined with DOX, HS-1200 (a synthetic chenodeoxycholic acid derivative), or lactacystin (a proteasome inhibitor).^304^ The synergy of VPA with TRAIL significantly enhanced apoptosis compared with TRAIL alone *in vitro*.^305^ Furthermore, vorinostat and VPA induced cell cycle arrest and raised PD-L1 expression in a patient-derived PF49 ATC cell line.^306^ In another scenario, VPA sensitized the 8505C ATC cell line to photon irradiation by diminishing DNA damage repair capacity.^307^ The clinical utility of VPA in ATC remains contentious and limited. A report highlighted significant tumor reduction (by 50.7% via CT measurement and 44.6% via ultrasound measurement) following combined oral VPA, cisplatin, and DOX chemotherapy, radiation, and surgery, sustaining a disease-free state for at least two years post-diagnosis.^308^ However, in an Italy-based multicenter randomized controlled phase II/III trial, the addition of VPA (1000 mg/day) to PTX (80 mg/m^2^/weekly) failed to improve progression-free survival or modulate PTX pharmacokinetics.^309^ The definitive role of VPA in ATC treatment remains uncertain.

#### Targeting TERT

Targeting TERT in ATC treatment needs more attention. Silencing of human TERT blocked ATC cell proliferation and migration significantly.^310^ Nanoparticles loaded human TERT siRNA showed tumor-suppression effect in ATC cell lines, and a similar effect was also observed *in vivo*.^311^ BIBR1532, a selective TERT inhibitor, induced G_0_/G_1_ cell cycle arrest and apoptosis in SW1736 ATC cell line.^312^ However, the value of TERT inhibition in treating ATC is yet to be evaluated deeply.

#### Targeting TP53

Unraveling the precise role of TP53 in ATC remains a priority, yet therapeutic interventions targeting TP53 hold promise in addressing this malignancy. Inhibiting TP53 has demonstrated efficacy in curbing cell proliferation. Herbimycin A, known for suppressing cell growth, reverses epithelial–mesenchymal transition by deactivating TP53 and PI3K-AKT signaling in the FRO cell line.^313^ Conversely, activating TP53 triggers apoptotic responses in ATC cells. Delivery of wild-type TP53 via adenovirus induces apoptosis,^314^ while apigenin fosters apoptosis in the FRO ATC cell line by augmenting c-myc levels and TP53 phosphorylation.^315^ Suberoyl bis-hydroxamic acid promotes apoptosis *in vivo* through the activation of the Notch1/TP53 signaling pathway.^316^ The combination of sorafenib and CP-31398, a TP53-restoring agent, effectively inhibits cell proliferation in the SW579 ATC cell line.^317^ Furthermore, modulating TP53 activity augments the efficacy of radiotherapy in ATC. Wild-type TP53 enhances the cytotoxic effects of NIS, heightening the accumulation of beta-emitter radionuclides and thereby enhancing radionuclide therapy.^318^ These approaches underscore the potential of TP53-targeted interventions in refining ATC management.

### Immunotherapy for ATC

#### Tumor immune microenvironment of ATC

Compared with other TC types, the tumor immune microenvironment of ATC is unique and somewhat mysterious. The microenvironment of most ATC was infiltrated by macrophages and CD8^+^ T cells. Compared with PTC, there exists more infiltration of exhausted CD8^+^ T cells and M2 macrophages and less cytotoxicity of CD8^+^ T cells, γδT cells, and natural killer (NK) cells in ATC, and the levels of immune checkpoint molecules (LAG-3, PD-1, HAVCR-2, and TIGIT) are also elevated.^319^ Compared with PDTC, the tumor proportion score of PD-L1 was elevated in ATC (7.7% *vs*. 60%; *P* = 0.006), and the amounts of CD3^+^ and CD8^+^ T cells, CD68^+^ and CD163^+^ macrophages, and S100^+^ dendritic cells were also elevated in ATC.^320^

#### Tumor-associated macrophages

TAMs emerge as pivotal actors in ATC pathogenesis, particularly in fostering metastasis. Pulmonary macrophages notably contribute to the pulmonary spread of ATC.^321^ Human ATC specimens exhibit robust infiltration of CD68^+^CD163^+^ TAMs,^322^ featuring ramified TAMs. These ramified TAMs intricately intermingle with ATC cells, forming a network through their ramifications, which extend from perivascular clusters and disperse within the tumor parenchyma.^323^ The abundance of TAMs inversely correlates with ATC prognosis, highlighting the potential of four TAM-related genes (FZD6, RBBP8, PREX1, HSD3B7) as potential biomarkers.^324^ A TAM-related prognostic index has been developed, displaying a positive association with TAM infiltration levels.^324^ Furthermore, CXCR4 expression significantly correlates with densities of CD163^+^ TAMs (*P* = 0.013).^325^

#### Immune genetic signature

The immune genetic signature of ATC is also yet to be explored deeply. CREB3L1 was identified as a key gene in ATC development and an upstream regulator of differentiation-related pathways (including epithelial–mesenchymal transition).^326^ Most immunogenic cell death genes were highly expressed in ATC, and five genes (TLR4, ENTPD1, LY96, CASP1, and PDIA3) were identified as the dynamic signature in the malignant progression of ATC.^327^ The T cell immunoglobulin and mucin-domain-containing protein-3 (TIM3) was identified as an immune checkpoint in macrophages,^328^ and TIM3 produced by ATC cells induced tumor-promoting M2-like macrophage polarization.^329^

### Immunotherapy for ATC

#### Targeting PD-1/PD-L1

PD-1/PD-L1 orchestrates immune tolerance within the tumor microenvironment, and its targeted inhibition has showcased considerable value in cancer treatment.^330^ Notably, most ATC cases exhibit positivity for PD-L1, whereas normal thyroid and DTC present with negative expression.^331^ Mean PD-L1 expression markedly elevates in ATC (tumor proportion score = 30%) compared with PDTC (tumor proportion score = 5%; *P* < 0.01) and normal thyroid tissue (tumor proportion score = 0%; *P* < 0.001).^332^ PD-L1 expression inversely correlates with the OS of individuals diagnosed with ATC.^333^ Differences in PD-L1 expression and lymphocyte infiltration distinguish advanced DTC from ATC.^334^ Elevated PD-1 expression in inflammatory cells significantly associates with poorer OS (HR = 3.36; 95%CI: 1.00–12.96; *P* < 0.05) in ATC.^335^

Immunotherapy's primary focus in ATC revolves around PD-1/PD-L1 targeting. Synergistic inhibition of primary ATC cell proliferation is observed with the combination of radiotherapy and atezolizumab (PD-L1 antibody).^336^ Moreover, dual inhibition of BRAF^V600E^ and PD-L1 leads to heightened local TAM levels and enhanced therapeutic nanoparticle delivery.^337^ Clinical studies and case reports collating immunotherapy efforts in ATC are summarized in Tables 5 and 6, respectively. These tables offer a comprehensive view of the clinical application of ATC immunotherapies. They underscore the predominant targeting of PD-1 in these therapies (except tremelimumab). Additionally, most immunotherapies in ATC are administered alongside targeted therapy and conventional trimodal therapy. However, the outcomes from clinical studies of immunotherapies (Table 5) are fewer compared with those of targeted therapies (Table 3, Table 2) in ATC. The development of immunotherapies for ATC remains restricted, warranting further exploration and advancement.

#### Other potential choices

Beyond the PD-1/PD-L1 focus, exploring other facets of ATC immunotherapy proves promising. Notably, two key areas show potential: targeting NK cells and delving into radioimmunotherapy. NK cells play a pivotal role in the ATC tumor microenvironment. Advanced TC patients, including those with ATC, exhibit an enrichment of CD56^hi^CD16^hi/lo^ NK cells. Compared with circulating CD56^lo^CD16^hi^ NK cells, CD56^hi^CD16^hi/lo^ NK cells demonstrate increased expression of CD158a and CD158b (inhibitory KIR family members) and decreased NKG2D (an NK cell activator).^338^ These CD56^hi^CD16^hi/lo^ NK cells exhibit higher PD-1 and TIM3 expression and diminished cytotoxicity against CAL-62 ATC cell lines. Dual blockade of PD-1 and TIM3 shows potential in boosting both CD56^hi^CD16^hi/lo^ and CD56^lo^CD16^hi^ NK cells from ATC patients.^338^ Additionally, NK cells have shown effectiveness in targeting pulmonary metastases of ATC *in vivo*,^339^ and ATC cell line inhibition was observed with UL16-binding proteins (ULBPs) 2/5/6, which attracted CXCR3^+^ NK cells.^340^

#### Inflammatory marker of ATC

The effective management of ATC is pivotal in curbing its mortality rates. To aid in this, several inflammatory biomarkers have surfaced as potential facilitators in managing ATC patients. These include the lymphocyte-to-monocyte ratio, neutrophil-lymphocyte ratio (NLR), platelet-to-lymphocyte ratio, and neutrophil-monocyte-platelet-to-lymphocyte ratio. Low lymphocyte-to-monocyte ratio levels have emerged as a marker linked to poorer OS among ATC patients.^341^ Similarly, NLR demonstrates a significant association with OS (HR = 3.18; 95%CI: 1.15–8.85; *P* = 0.026), with noticeable differences in OS curves concerning post-radiotherapy NLR (*P* = 0.036).^342^ Neutrophil-monocyte-platelet-to-lymphocyte ratio stands out as an independent predictor for the OS of both ATC and advanced DTC patients (HR = 6.470; 95%CI: 2.134–19.616; *P* = 0.001).^343^ Notably, ATC patients experiencing an increase in NLR from their baseline values exhibit a worse prognosis compared with those without such elevation.^344^ However, baseline values of NLR, platelet-to-lymphocyte ratio, and lymphocyte-to-monocyte ratio seem to show no significant differences in OS.^344^ Despite these observations, the precise value and utility of these inflammatory biomarkers in the context of ATC management await further determination.

## Discussion

### Single-cell RNA sequencing in ATC

In recent years, experimental studies of ATC have been empowered by single-cell RNA sequencing techniques. Besides the genetic landscape and anaplastic transformation mentioned above,^6^ more features of ATC were revealed. ATC cells showed resistance to DNA damages from γ-radiation by activating genes associated with homologous recombination and non-homologous end joining,^345^ and hyper-activation of one-carbon metabolism was observed in the transformation from PTC to ATC.^346^ Interferon-stimulated gene 15 (ISG15) correlated significantly with the proliferation and malignancy of the ATC cancer stem cells.^347^ SIGLEC15 deactivated T cells by blocking NFAT1, NFAT2, and NF-κB signaling pathways, and SIGLEC15 inhibition stimulated the secretion of IFN-γ and IL-2.^348^ More subtypes of tumor-infiltrating lymphocytes have been gradually identified. One ATC-specific ATC-associated macrophage subgroup, IL2RA^+^VSIG4^+^ TAMs, was identified and associated with the better prognosis of ATC patients.^349^ CXCL13^+^ T cells and early tertiary lymphoid structure facilitated the immunotherapy for ATC.^350^ Future research into ATC requires more extensive application of the single-cell RNA sequencing technique, and the value of spatial RNA sequencing technique, which is rarely deployed in dissecting ATC presently, remains to be examined in the pathogenesis and progression of ATC.

### Diagnosis of ATC

Diagnosis of ATC comprises two essential components, invasive tissue sampling, yielding cytological and pathological evidence supported by immunohistochemistry, and imaging modalities, with ^18^F-fluorodeoxyglucose (FDG) positron emission tomography/computed tomography (PET/CT) playing a central role in accurate staging.^175^^,^^351^

### Invasive tissue sampling

FNA and core needle biopsy (CNB) represent standard minimally invasive tissue sampling techniques.^352^ FNA has revealed several critical cytological features of ATC including nuclear pleomorphism, coarse/clumped chromatin, macro-nucleoli, apoptosis, and necrosis.^353^ FNA shows an accuracy rate of 86.5% in diagnosing a cohort of 163 ATC cases,^354^ with initial ultrasonography-guided FNA achieving a correct diagnosis of ATC in 50% of cases.^355^ However, the effectiveness of FNA encounters challenges from CNB. In a cohort of 59 ATC cases, CNB shows a higher sensitivity of 87.5% and a positive predictive value of 100.0% for diagnosing ATC than FNA (50.6% and 90.9%, respectively).^356^ The rate of diagnostic surgery is significantly lower after CNB (12.5%) than after FNA (35.4%) (*P* = 0.020).^356^ Similarity, a meta-analysis reported that CNB showed a higher sensitivity (80.1%) value for diagnosing ATC than that of FNA (61%) and exhibited a positive predictive value of 100% for ATC.^357^ Meanwhile, the need for additional diagnostic surgery after CNB was 17.6% for ATC.^357^ Sensitivity and specificity of both FNA and CNB in diagnosing ATC need more independent exploration and validation.

### Immunohistochemistry

Immunohistochemistry plays a crucial role in establishing the diagnosis of ATC through tissue sampling. Comparative analyses with PTC reveal significantly elevated expression of cancer stem cell markers in ATC, notably chemoresistance markers, which correlate with diminished overall survival in ATC cases.^358^ Moreover, immunohistochemical profiling facilitates the identification of an 8-marker transformation panel that exhibits 100% accuracy, sensitivity, and specificity in distinguishing ATC from DTC.^359^

Two immunohistochemical biomarkers in ATC are worthy of note. One is paired box gene 8 (PAX8). It was reported that all three FNA samples of ATC were PAX8 positive.^360^ Positive PAX8-staining is reported in five of seven ATC cases mimicking primary head and neck squamous cell carcinoma.^361^ PAX8 expression was positively correlated with an epithelial pattern (*P* = 0.0008),^362^ a coexisting differentiated thyroid carcinoma component (*P* = 0.0004),^362^ and improved OS (*P* = 0.019).^363^ Meanwhile, another study reported that PAX8 staining was positive in 26 (76%) ATC cases, including all 16 squamodisc variants, 7 (58%) giant cell/pleomorphic variants, and 3 (50%) spindled variants, and all head and neck squamous cell carcinomas were negative for PAX8 contrastly.^364^ Three immunohistochemical features of ATC are proposed: β-catenin nuclear expression with no or reduced cell membranous expression, the loss or discontinuous pattern of E-cadherin expression, and the loss of PAX8 nuclear expression.^365^ However, the sensitivity and specificity of PAX8 in diagnosing ATC need more improvement. In a cohort comprising 6 cases of ATC, all exhibited positive staining for pan-cytokeratin, but PAX8 expression was detected in only 40% of these cases.^366^ In another cohort of 29 ATC cases, the detection rates for thyroid transcription factor-1 (TTF-1), PAX8, and E-cadherin were 17.2%, 51.7%, and 10.3%, respectively.^365^ Prostate-specific membrane antigen (PSMA), the other immunohistochemical biomarker in ATC, needs more attention. Six of the eight analyzed patients (2 ATCs and 4 PDTCs) showed increased glucose metabolism without increased PSMA uptake after PET/CT, while immunohistochemical analysis of PSMA expression in corresponding patient tissue samples reported that there was strong PSMA expression in 27 of the analyzed 39 ATC and 13 of the analyzed 22 PDTC tissue sections.^367^ There was a correlation between immunohistochemical PSMA expression and uptake on gallium-68 (^68^Ga)-PMSA-PET/CT in three of the examined patients.^367^ In spite of that, the role of PSMA in PET/CT imaging is controversial in ATC. Although ^68^Ga-PMSA-PET/CT demonstrated a lower detection rate (3/11) than FDG-PET/CT (8/11) when visualizing TC lesions (total of 11 ATC cases),^368^ it was also reported that ^68^Ga-PMSA-PET/CT showed high uptake in the primary tumor, cervical, and mediastinal nodes in an ATC case.^369^

To date, there remains a need for further exploration into the breadth and effectiveness of immunohistochemical biomarkers for diagnosing ATC. Additionally, unlocking the full potential of immunohistochemistry in elucidating pathological characteristics and monitoring disease progression in ATC is imperative.

### ^18^F-FDG PET/CT

^18^F-FDG PET/CT plays a pivotal role in assessing tumor progression and tailoring disease management strategies for ATC patients.^175^^,^^351^ ATC demonstrates robust uptake on ^18^F-FDG PET images, significantly influencing the clinical management of half of the ATC cohort (16 ATC cases).^370^ Various PET parameters, including elevated maximum standardized uptake value (SUV_max_), metabolic tumor volume, and total lesion glycolysis, are closely associated with adverse prognosis (*P* < 0.001, *P* = 0.002, and *P* < 0.001, respectively).^371^ While variations in SUV_max_ and occurrences of local relapse exhibit no significant correlation potentially due to the limited availability of assessable ^18^F-FDG PET/CT ATC cases (less than 50%),^372^ both the volume (≥300 mL) and intensity (SUV_max_ ≥18) of FDG uptake emerge as significant prognostic indicators for ATC patient survival.^373^ The comprehensive assessment of ^18^F-FDG PET/CT in diagnosing ATC is elucidated in Table 8, underscoring its considerable diagnostic utility. The future clinical utility of ^18^F-FDG PET/CT holds promise for monitoring therapeutic efficacy,^374^ paving the way for expanded applications in ATC management.

**Table 8:** Case reports about applying^18^F-FDG PET/CT in diagnosing anaplastic thyroid cancer.

Reference	Age (years)	Gender	Imaging findings in diagnosis	Oncological features caught by imaging
Iagaru et al^451^	51	Female	Multiple pulmonary metastases and a left adrenal lesion	Adrenal metastasis
Nguyen et al^452^	76	Female	i) High metabolic large masses in the right neck; ii) lower neck near the midline extending to the upper mediastinum; iii) large lower neck/mediastinal mass compressing the trachea	Tumor staging and evaluation after therapy
Strobel et al^453^	46	Male	i) “Worm-like” increased FDG uptake extending from the primary tumor into the mediastinum and ending just above the right atrium; ii) FDG-active lesion is located within the dilated superior vena cava	Vascular tumor invasion
Zweifel et al^454^	57	Male	Increased^18^F-FDG uptake in the cervical/retrosternal mass, in the bone marrow, and in the enlarged spleen	Bone and spleen metastasis
Yurkiewicz et al^455^	61	Female	Extensive hypermetabolic lesions throughout the skeletal musculature concerning metastatic disease	Skeletal muscle metastasis

Note: ^18^F-FDG, ^18^F-fluorodeoxyglucose positron emission tomography/computed tomography.

### Prognosis of ATC

Prognosis is a direct indicator of treatment efficacy, and challenges in the treatment of ATC can be characterized by independent prognostic factors for their pivotal role in assessing conditions, guiding treatment decisions, and enhancing survival outcomes. Several clinical studies of independent prognostic factors in ATC have been conducted,^375^, ^376^, ^377^, ^378^, ^379^, ^380^, ^381^, ^382^, ^383^, ^384^, ^385^, ^386^, ^387^, ^388^, ^389^ and they have been summarized in Table 9, Table 10, Table 11, Table 12, Table 13, Table 14, Table 15, Table 16, Table 17. Independent prognostic factors of ATC in these tables can be categorized into three main domains: patient's initial condition, tumor staging, and therapeutic interventions. Subsequent discussion will delve into these domains to reveal the challenges in fighting with ATC.

**Table 9:** Age as an independent prognostic factor of anaplastic thyroid cancer (*P* < 0.05).

Reference	Cases	Independent prognostic factor		HR	95%CI	*P*-value
Jergin et al^375^	261	Age of diagnosis		1.02	1.00–1.03	0.007
Junko et al^376^	100	Age		1.03^a^	1.01–1.05	0.014
Zivaljevic et al^389^	150	Patient age		0.68^b^	0.49–0.95	0.023
de Ridder et al^383^	812	Age		1.014	1.006–1.020	<0.001
Wu et al^385^	97	Age at diagnosis		1.03	1.01–1.06	Significant but not available
Hvilsom et al^381^	219	Older age		1.4	1.0–2.0	Significant but not available
Wendler et al^379^	100	Age at initial diagnosis	<70 years	Ref		
			≥70 years	1.048	1.015–1.082	0.004
Glaser et al^380^	3552	Age	<65 years	Ref		
			≥65 years	1.42	1.26–1.60	<0.0005
Zhou et al^387^	491	Age	<65 years	Ref		
			≥65 years	1.31^c^	1.07–1.62	0.011

Note:

a HR is unavailable and replaced by risk ratio in the original.

b HR is unavailable and replaced by the odd ratio in the original.

c HR is adjusted by the inverse probability weighting for balancing variables between groups. CI, confidential interval; HR, hazard ratio; Ref, reference category.

**Table 10:** Clinical presentations as independent prognostic factors of anaplastic thyroid cancer (*P* < 0.05).

Reference	Cases	Independent prognostic factor		HR	95%CI	*P*-value
Junko et al^376^	100	Leukocytosis (white blood cell count ≥10,000/mm^3^)		2.04^a^	1.26–3.24	0.004
Hvilsom et al^381^	219	Respiratory impairment at diagnosis		2.0	1.2–2.6	Significant but not available
Hvilsom et al^381^	219	Vocal fold palsy at diagnosis		1.1	0.8–1.6	
Jannin et al^384^	295	Neutrophil-lymphocyte ratio	<5.05	Ref		
			≥5.05	2.05	1.39–3.03	<0.00
Sun et al^377^	60	White blood cell counts	<10.0 × 10^9^/L	Ref		
			≥10.0 × 10^9^/L	1.869^b^	1.069–3.269	0.028
Glaser et al^380^	3552	Charlson–Deyo comorbidity score	0	Ref		
			1	1.36	1.19–1.55	<0.0005
			≥2	1.69	1.33–2.14	

Note.

a HR is unavailable and replaced by risk ratio (RR) in the original.

b HR is adjusted by age, white blood cell count, distant metastasis, clinical tumor-node-metastasis stage, chemotherapy, radiotherapy, and therapeutic regimen. CI, confidential interval; HR, hazard ratio; Ref, reference category.

**Table 11:** Primary tumor (T) as an independent prognostic factor of anaplastic thyroid cancer (*P* < 0.05).

	Reference	Cases	Independent prognostic factor		HR	95%CI	*P*-value
T status	Hvilsom et al^381^	219	T4b		1.6	1.0–2.6	Significant but not available
Extent of primary disease	Glaser et al^380^	3552	Primarily confined to the thyroid	Yes	Ref		
				No/unknown	1.36	1.13–1.62	0.001
	Jergin et al^375^	261	Extent of primary disease	Confined	Ref		
				Extracapsular extension	1.68	1.05–2.70	0.032
				Further extension or metastasis	3.64	2.23–5.94	<0.0001
Tumor size	Liu et al^386^	50	Diameter of primary tumor ≤4 cm		0.264^a^	Not available	0.001
	Glaser et al^380^	3552	Tumor size	≤6 cm	Ref		
				>6 cm	1.36	1.23–1.55	<0.0005
	Jergin et al^375^	261	Tumor size	≤7 cm	Ref		
				>7 cm	1.59	1.05–2.70	0.010
				Unknown	1.51	1.09–2.10	0.014
Extrathyroidal invasion	Junko et al^376^	100	Extrathyroidal invasion		3.02^b^	1.17–10.39	0.021
	Mohebati et al^378^	83	Gross extrathyroidal extension	No	Ref		
				Yes	2.293	1.5–5.8	0.002
	Zhou et al^387^	491	Tumor extension	I	Ref		
				IV	1.64^c^	1.17–2.30	0.004

Note:

a HR is unavailable and replaced by odd ratio in the original.

b HR is unavailable and replaced by the risk ratio in the original.

c HR is adjusted by the inverse probability weighting for balancing variables between groups. CI, confidential interval; HR, hazard ratio; Ref, reference category.

**Table 12:** Lymph node metastasis (N) as an independent prognostic factor of anaplastic thyroid cancer (*P* < 0.05).

Reference	Cases	Independent prognostic factor		HR	95%CI	*P*-value
Glaser et al^380^	3552	Nodal classification	Clinically or pathologically positive	Ref		
			Negative/unknown	0.81	0.72–0.90	<0.0005
de Ridder et al^383^	812	N status	N_0_	Ref		
			N_+_	1.2	1.0–1.4	0.020
			N_x_	1.2	1.0–1.5	0.050

Note: CI, confidential interval; HR, hazard ratio; Ref, reference category.

**Table 13:** Distant metastasis (M) as an independent prognostic factor of anaplastic thyroid cancer (*P* < 0.05).

Reference	Cases	Independent prognostic factor		HR	95%CI	*P*-value
Junko et al^376^	100	Distant metastasis		1.94^a^	1.18–3.25	0.009
Liu et al^386^	50	Distant metastasis		3.438^b^	Not available	0.002
Hvilsom et al^381^	219	Distant metastases		2.7	1.8–3.9	Significant but not available
Wendler et al^379^	100	M status	M_0_	Ref		
			M_1_	2.718	1.384–5.342	0.004
de Ridder et al^383^	812	M status	M_0_	Ref		
			M_1_	1.8	1.5–2.1	<0.001
Zhou et al^387^	491	Distant metastasis	M_0_	Ref		
			M_1_	1.87^c^	1.52–2.30	<0.001

Note:

a HR is unavailable and replaced by risk ratio in the original.

b HR is unavailable and replaced by the odd ratio in the original.

c HR is adjusted by the inverse probability weighting for balancing variables between groups. CI, confidential interval; HR, hazard ratio; Ref, reference category.

**Table 14:** TNM staging as an independent prognostic factor of anaplastic thyroid cancer (*P* < 0.05).

Reference	ATC cases	Independent prognostic factor		HR	95%CI	*P*-value
Wu et al^385^	97	Stage IVC		2.65	1.35–5.18	Significant but not available
Simões-Pereira et al^382^	79	Stage at diagnosis	Stage IVA	Ref		
			Stage IVC	3.327^a^	1.001–11.055	0.050
Jannin et al^384^	295	Stage	Stage IVB	Ref		
			Stage IVC	1.78	1.33–2.51	<0.001

Note:

a HR is unavailable and replaced by the odd ratio in the original. CI, confidential interval; HR, hazard ratio; Ref, reference category.

**Table 15:** Surgery as an independent prognostic factor of anaplastic thyroid cancer (*P* < 0.05).

	Reference	Cases	Independent prognostic factor		HR	95%CI	*P*-value	
Decision about undergoing surgery	Junko et al376	100	No surgical resection		3.99^a^	2.37–6.66	<0.0001	
	Zivaljevic et al^389^	150	Surgical intervention		0.43^b^	0.29–0.63	0.000	
	Liu et al^386^	50	Surgery		0.331^b^	Not available	0.038	
	Yamazaki et al^388^	66	Resection	No	Ref			
				Yes	0.316	0.129–0.773	0.012	
Surgical method	Wendler et al^379^	100	Thyroid surgery	Radical	Ref			
				other or none	2.201	1.186–4.086	0.012	
	Glaser et al^380^	3552	Surgery	Total thyroidectomy	Ref			
				Other surgery	1.32	1.13–1.54	<0.0005	
				None	1.76	1.52–2.03		
	Zhou et al^387^	491	Surgery	No	Ref			
				Non-thyroidectomy	0.68^c^	0.53–0.89	0.004	
				Thyroidectomy	0.51^c^	0.40–0.66	<0.001	
Surgical scope	Mohebati et al^378^	83	Resection type	R_0_/R_1_	Ref			
				R_2_/R_x_	2.021	1.0–3.9	0.037	
	Wendler et al^379^	100	Complete local resection	Yes	Ref			
				No	5.539	1.858–16.514	0.002	
	Glaser et al^380^	3552	Surgical margins	Negative	Ref			
				positive/unknown	1.46	1.21–1.77	<0.0005	

Note:

a HR is unavailable and replaced by risk ratio in the original.

b HR is unavailable and replaced by the odd ratio in the original.

c HR is adjusted by the inverse probability weighting for balancing variables between groups. CI, confidential interval; HR, hazard ratio; Ref, reference category.

**Table 16:** Chemotherapy as an independent prognostic factor of anaplastic thyroid cancer (*P* < 0.05).

Reference	Cases	Independent prognostic factor		HR	95%CI	*P*-value
Liu et al^386^	50	Chemotherapy		0.173^a^	Not available	0.003
Wendler et al^379^	100	Chemotherapy	No	Ref		
			Yes	11.636	2.424–60.394	0.003
Glaser et al^380^	3552	Chemotherapy	Yes	Ref		
			No	1.32	1.16–1.50	<0.0005
Yamazaki et al^388^	66	Response to paclitaxel	No	Ref		
			Yes	0.423	0.193–0.930	0.032

Note:

a HR is unavailable and replaced by the odd ratio in the original. CI, confidential interval; HR, hazard ratio; Ref, reference category.

**Table 17:** Radiotherapy as an independent prognostic factor of anaplastic thyroid cancer (*P* < 0.05).

Reference	Cases	Independent prognostic factor		HR	95%CI	*P*-value
Junko et al^376^	100	Radiation <40 Gy		2.96^a^	1.86–4.72	<0.0001
Wu et al^385^	97	Absence of definitive or adjuvant radiotherapy		1.90	1.01–3.59	Significant but not available
Liu et al^386^	50	Radiotherapy		0.297^b^	Not available	0.007
Wendler et al^379^	100	External beam radiotherapy	≥40 Gy	Ref		
			<40 Gy	0.339	0.152–0.759	0.008
Yamazaki et al^388^	66	Radiotherapy	No	Ref		
			Yes	0.229	0.100–0.526	0.001
Zhou et al^387^	491	Treatment	Radiotherapy alone	Ref		
			Radiotherapy plus chemotherapy	0.69^c^	0.56–0.85	<0.001
Glaser et al^380^	3552	Radiotherapy	None	Ref		
			36.1–59.3 Gy	0.58	0.50–0.69	<0.0005
			≥59.4 Gy	0.41	0.35–0.49	

Note:

a HR is unavailable and replaced by risk ratio in the original.

b HR is unavailable and replaced by the odd ratio in the original.

c HR is adjusted by the inverse probability weighting for balancing variables between groups. CI, confidential interval; HR, hazard ratio; Ref, reference category.

### Patient's initial condition

#### Age

Age at diagnosis basically correlates with inferior OS among patients with ATC (Table 9). Age at diagnosis exceeding 70 years amplifies the risk among ATC patients (HR = 1.662; 95%CI: 1.321–2.092), with a substantial disparity observed in cancer-specific mortality rates per 1000-person-years between individuals younger and older than 70 years (949.980 (95%CI: 827.323–1090.822) *vs*. 1546.667 (95%CI: 1333.114–1794.428); *P* < 0.001).^390^ Nowadays, more attention should be given to the early screening of ATC to decrease the risk of ATC patients, and the classification of ATC patients may facilitate ATC management, as shown by a valuable tool for risk stratification based on age in forecasting the outcomes of ATC patients.^391^

#### Clinical presentation

Table 10 details the prognostic role of clinical presentation, which includes experimental examination, complications, comorbidities, and daily living abilities. This tabulation shows that clinical presentation is controversial for treating ATC.

On the one hand, experimental examination of suspicious people can be practical in early-stage screening, diagnosis, and modifying therapeutic schemes. Leucocytosis and NLR have been identified as correlating with inferior OS among ATC patients (Table 10). More independent research on the value of experimental examination is warranted.

On the other hand, complications at diagnosis call for more active interventions for their opposing roles in the prognosis of ATC. Three complications, respiratory impairment (Table 10), vocal fold palsy (Table 10), and dyspnoea,^392^ have portend a poorer prognosis. Additionally, comorbidities, as assessed by the Charlson–Deyo comorbidity score, exert a negative effect on prognosis (Table 10). Patients' complications and comorbidities should be appropriately evaluated and handled when facing therapeutic options for ATC, and the burden of ATC patients can be lightened extensively.

#### Tumor staging

TNM staging is essential in delineating the anatomical extent of ATC and establishing its stage to guide tailored treatment strategies. It consists of three sections, primary tumor (T), lymph nodes metastasis (N), and distant metastasis (M).^393^

The value of three TNM sections in determining prognosis independently has been examined initially. Primary tumors (T status), as detailed in Table 11, can be characterized by tumor size, the extent of primary disease, and extrathyroidal invasion (extension). Larger tumor size and extrathyroidal invasion (extension) are correlated with reduced OS among ATC patients, whereas a confined extent of primary disease is associated with improved OS outcomes. Lymph node metastasis (N Status), as detailed in Table 12, also showcases its independence in determining the prognosis of ATC patients. Nodal classification as negative/unknown improves OS outcomes, whereas N status as N_+_/N_x_ indicates decreased OS among ATC patients. A retrospective study (*n* = 313) reported that lymph node metastasis emerges as an independent risk factor for ATC mortality (adjusted HR = 1.47; 95%CI: 1.10–1.96; *P* = 0.009).^394^ Distant metastasis (M Status), as detailed in Table 13, correlates with impaired OS, and there exists a significant difference between the whole ATC cohort (*n* = 152) and ATC with distant metastasis groups (*n* = 88) within the whole cohort in the mortality (76% *vs*. 90%; *P* = 0.01), survival >1 year (32% *vs*. 15%; *P* = 0.003), and median survival (228.5 *vs*. 171 days; *P* = 0.01).^395^

Each section of TNM staging can independently influence the prognosis of ATC, and based on the assessment of all sections, ATC cases will be classified into stage IVA, stage IVB, or stage IVC.^393^ Generally, a higher stage denotes a graver risk of ATC patient survival, as outlined in Table 14. Challenges to the precise staging of ATC are still worthy of consideration and solution, and dynamic monitoring of ATC progression urges further investigations on the exact results and prognostic value of three sections of TNM staging.

#### Therapeutic interventions

The efficacy and limitations of trimodal therapy, comprising surgery, chemotherapy, and external beam radiotherapy, have been discussed earlier. Recently, each part of trimodal therapy has been gradually scrutinized for its independence in influencing the prognosis of ATC, and the result from such scrutinization directs the future optimization of ATC therapy.

#### Surgery

Independent impact of surgery on prognosis is outlined in Table 15. Although the decision to undergo surgery correlates with improved OS of ATC patients, a pooled analysis exhibited that surgery brings higher risk to ATC patients (HR = 1.997; 95%CI: 1.162–3.433; *P* = 0.012).^396^ Maximum surgical scope and negative surgical margins indicate the prolonged OS of ATC patients. In a retrospective study of 233 stage IVB ATC patients, the super-radical resection group (*n* = 23) received an improved one-year cause-specific survival rate compared with the no/palliative surgery group (*n* = 80 and 72, respectively) (*P* = 0.0065).^397^

Although surgery has been strongly recommended for stage IVA and resectable stage IVB ATC patients,^175^ more effort to extend and ameliorate the application of surgery is warranted. To begin with, increasing the opportunity and wish for undergoing surgery is vital for the initial treatment of ATC. ATC patients without thyroid resections have older age and more advanced stage compared with surgical patients (both *P* < 0.001).^398^ Besides, a thyroidectomy should be regularly performed to facilitate further treatment and improve OS. Finally, radical resection and negative margin should be sought and combined with other therapies for better clinical outcomes. Negative margin status was more often achieved in stage IVA ATC patients (*P* < 0.001), and positive margin status was associated with higher mortality in stage IVA ATC patients (*P* = 0.017) but had no influence on the survival of stages IVB and IVC (*P* > 0.05).^398^

#### Chemotherapy

The role of chemotherapy in the prognosis of ATC seems confusing, as shown in Table 16. Despite the efficacy of and sensitivity to chemotherapy in improving OS, some observations show its effect on increasing the risk of further survival of ATC patients. A meta-analysis also reported that chemotherapy did not prolong the survival of ATC patients compared with controls (HR = 0.63; 95%CI: 0.33–1.21; *Z* = 1.39; *P* = 0.17).^399^ The therapeutic effect of chemotherapy demands independent validations and personalized chemotherapeutic options should be advised for desirable clinical outcomes.

#### Radiotherapy

Table 17 outlines the prognostic role of radiotherapy, which shows the significant reduction in the risk of ATC patients by radiotherapy. Radiotherapy is a default option for all stages of ATC patients,^175^ and the combination of radiotherapy and surgery also showcases excellent benefits. One meta-analysis also reported the combination of surgery and radiotherapy significantly reduced the risk of death compared with surgery alone (HR = 0.51; 95%CI: 0.36–0.73; *Z* = 3.66; *P* = 0.0002) for resectable ATC cases,^399^ and another meta-analysis reported that postoperative radiotherapy significantly reduced the risk of death in all the patients with resected ATC compared with those with surgery alone (HR = 0.556; 95%CI: 0.419–0.737; *P* < 0.001).^400^

Despite that, more effort is warranted in the optimization of radiotherapy. On the one hand, higher radiation dose correlates with improved OS. A reasonable elevation of radiation dose is necessary for better clinical outcomes, and side effects deserve attention. It was reported that radiation dose ≥50 Gy was associated with less dysphagia (odd ratios (OR) = 0.2; 95%CI: 0.05–0.9; *P* = 0.029).^401^ On the other hand, the optimal technique for administering radiotherapy needs examination. Besides external beam radiotherapy, one radiation delivery technique, intensity-modulated radiotherapy or volumetric modulated arc therapy, correlates with lower skin toxicity (OR = 0.2; 95%CI: 0.04–0.9; *P* = 0.045).^401^ Comparison among various radiotherapeutic techniques is essential for effective ATC treatment.

## Conclusion

**Figure 1:**
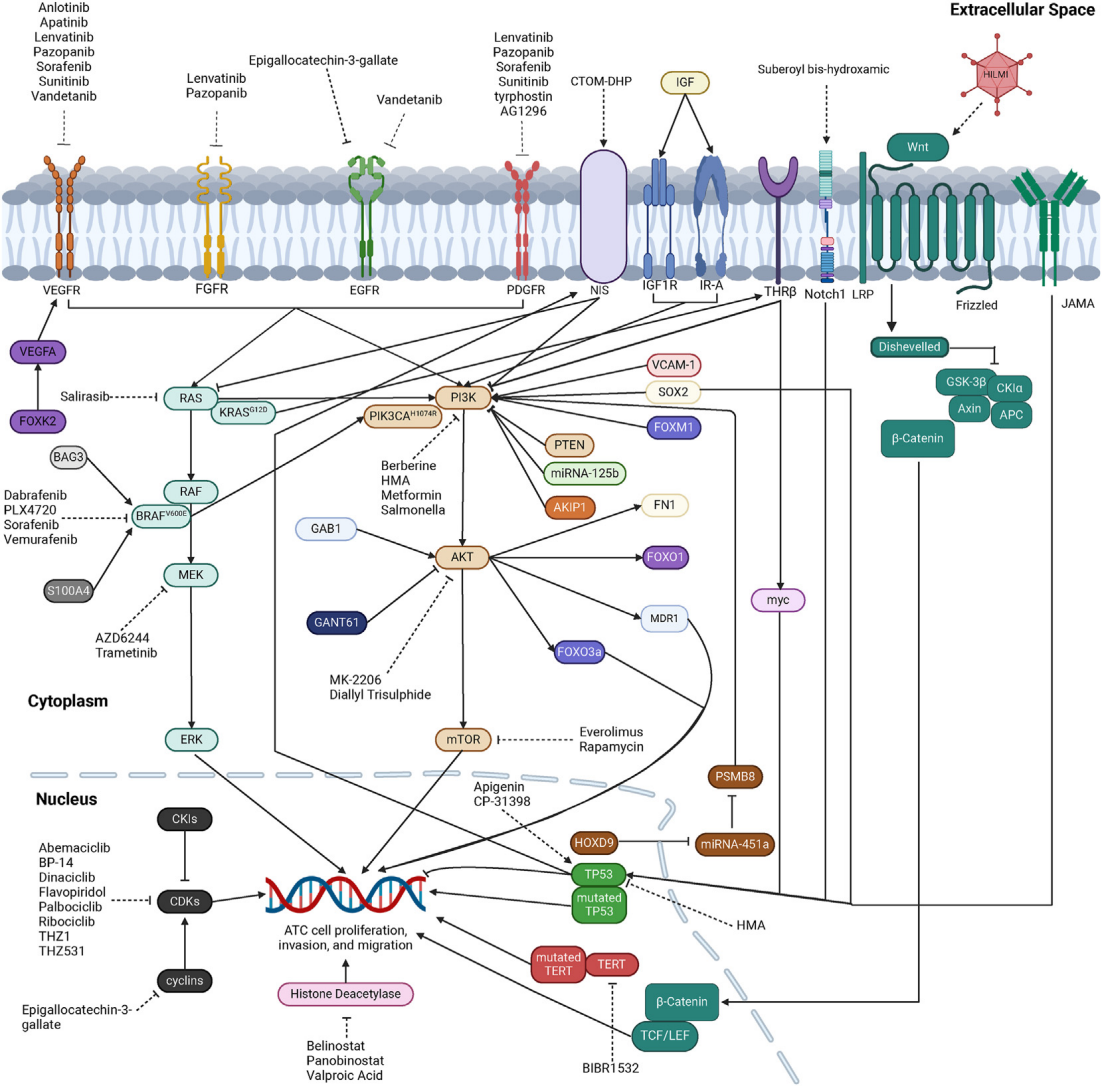
Key signaling pathways in anaplastic thyroid cancer. Different pathways are labeled in different colors, and different components in the same pathway are portrayed in the same color. This picture is created with BioRender.com.

ATC, the most aggressive form of TC, poses significant challenges due to its unclear pathogenesis. Key signaling pathways, namely the MAPK and PI3K-AKT-mTOR pathways (Fig. 1), play pivotal roles in ATC tumorigenesis. Known molecular drivers such as KRAS^G12D^ and BRAF^V600E^ mutations contribute substantially to this process. Next-generation sequencing of ATC samples (detailed in Table 1) has unveiled additional gene aberrations in ALK, CDK, TERT, TP53, and Wnt pathways, all crucial in regulating cell proliferation and homeostasis. These mutations foster the immortality and invasiveness of ATC cells. Additionally, dysfunction of mitochondrial metabolism accelerates ATC tumorigenesis, and mitochondrion-target therapies have been gradually allocated with adequate attention, especially their synergistic effect with chemotherapy for ATC.

Targeted therapies, outlined in Table 2, Table 3, Table 4, have supplemented conventional trimodal therapies for ATC. Agents targeting RTKs (like lenvatinib and sorafenib) and RAF (notably the dabrafenib–trametinib combination) are under scrutiny for their potential responsiveness and potential to enable curative approaches. However, the efficacy and safety of other agents in targeted therapies require more independent clinical studies. While approaches targeting the PI3K-AKT-mTOR cascade exhibit diversity, they largely remain in the experimental phase. Among alternative targeted therapies, CDKs and histone deacetylase inhibitors hold promises for future clinical applications.

**Figure 2:**
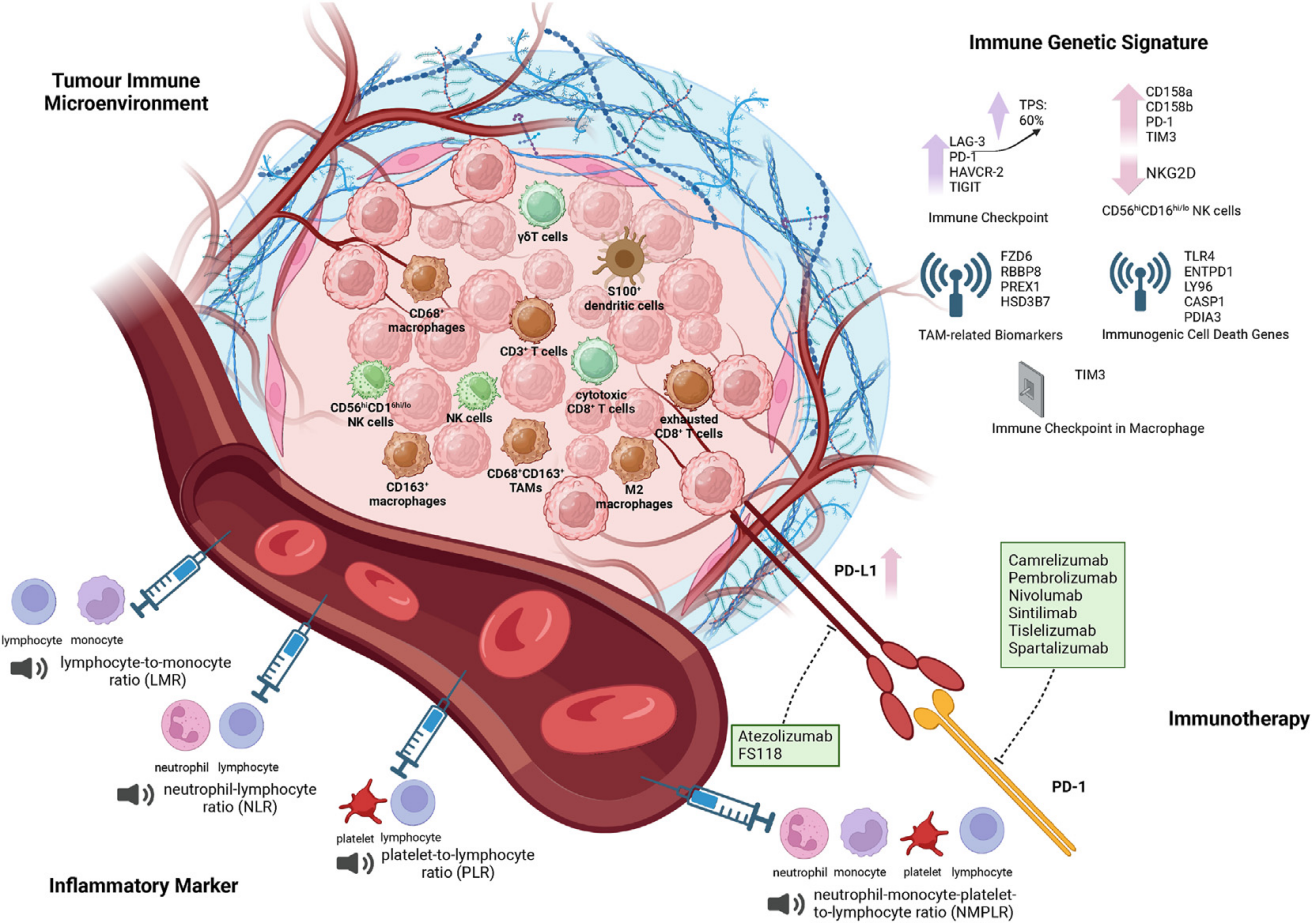
The tumor immune microenvironment of, immunotherapy for, and inflammatory marker for anaplastic thyroid cancer. This picture is created with BioRender.com.

Distinct differences in the tumor immune microenvironment distinguish ATC from other TC types. Dysregulated PD-1/PD-L1 expression and their close correlation with clinical outcomes have spurred the exploration and trials of ATC immunotherapy, detailed in Table 5, Table 6. The amalgamation of immunotherapy, targeted therapy, and conventional treatment could be pivotal in the management of ATC. Beyond PD-1/PD-L1 targeting, experimental approaches involving NK cell targeting and radioimmunotherapy offer innovative avenues. Leveraging inflammatory markers promises rigorous evaluation and precise management of ATC. A brief graphic summary of the immune microenvironment of and immunotherapy for ATC is provided in Figure 2. Noteworthily, the globally ongoing clinical trials were collected from ClinicalTrials.gov and listed in Table 7, and we hope that novel therapeutic options can be available from these clinical trials.

The diagnosis of ATC is based on invasive tissue sampling and imaging modalities. Although FNA reveals the unique cytological features of ATC, CNB exhibits its potential to replace FNA with higher sensitivity and 100% positive predictive value. Their value in diagnosing ATC requires further exploration. Immunohistochemistry showcases its diagnostic value by disclosing potential biomarkers for ATC, and the sensitivity and specificity of immunohistochemical biomarkers are worthy of examination and validation. As the recommended imaging modality of ATC, ^18^F-FDG PET/CT can detect the unique oncological features of ATC, as shown in Table 8, and several parameters of ^18^F-FDG PET/CT also correlate with inferior OS, indicating the promising future in deploying ^18^F-FDG PET/CT for panels specialized in ATC.

Prognosis directly indicates treatment efficacy, and independent prognostic factors, detailed in Table 9, Table 10, Table 11, Table 12, Table 13, Table 14, Table 15, Table 16, Table 17, reveal the ATC treatment's status quo and limitations. To begin with, the patient's initial condition should be valued. Early screening of suspicious people is necessary for reducing the risk of older age, and age should be considered when classifying ATC patients and administering personalized treatment. Clinical presentations of ATC patients should also be handled actively. Experimental examination of specific biomarkers can be developed for early screening and diagnosis, and patients' complications and comorbidities should be assessed and controlled for better clinical outcomes. Moreover, precise evaluation of TNM staging is the cornerstone of considering therapeutic options and dynamic monitoring of ATC progression. Finally, the value of trimodal therapy, the default therapeutic option, is acknowledged fully. However, improvements in trimodal therapy are encouraged to lessen the burden and ameliorate the OS of ATC patients, such as the extension of thyroidectomy, validation and exploration of chemotherapy, and augmented doses of radiotherapy. Combining trimodal therapy and new therapy (targeted therapy and immunotherapy) deserves rigorous evaluation and broadening applications.

## CRediT authorship contribution statement

**Zhao Zou:** Data curation, Formal analysis, Investigation, Methodology, Visualization, Writing – original draft, Writing – review & editing. **Linhong Zhong:** Conceptualization, Supervision, Validation.

## Conflict of interests

The authors have no competing interests to declare.
